# A Digital Educational Intervention With Wearable Activity Trackers to Support Health Behaviors Among Childhood Cancer Survivors: Pilot Feasibility and Acceptability Study

**DOI:** 10.2196/38367

**Published:** 2022-08-17

**Authors:** Lauren Ha, Claire E Wakefield, David Mizrahi, Claudio Diaz, Richard J Cohn, Christina Signorelli, Kalina Yacef, David Simar

**Affiliations:** 1 School of Health Sciences UNSW Medicine and Health UNSW Sydney Sydney Australia; 2 Kids Cancer Centre Sydney Children's Hospital Randwick Australia; 3 School of Clinical Medicine Discipline of Paediatrics and Child Health UNSW Sydney Sydney Australia; 4 Prince of Wales Clinical School UNSW Medicine and Health UNSW Sydney Sydney Australia; 5 The Daffodil Centre The University of Sydney, a joint venture with Cancer Council NSW Sydney Australia; 6 School of Computer Science Faculty of Engineering The University of Sydney Sydney Australia

**Keywords:** childhood cancer, survivorship, physical activity, exercise, activity tracker, eHealth, education, behavior change

## Abstract

**Background:**

Childhood cancer survivors are at increased risk of cardiometabolic complications that are exacerbated by poor health behaviors. Critically, many survivors do not meet physical activity guidelines.

**Objective:**

The primary aim was to evaluate the feasibility and acceptability of *iBounce*, a digital health intervention for educating and engaging survivors in physical activity. Our secondary aims were to assess the change in survivors’ physical activity levels and behaviors, aerobic fitness, and health-related quality of life (HRQoL) after participating in the iBounce program.

**Methods:**

We recruited survivors aged 8 to 13 years who were ≥12 months post cancer treatment completion. The app-based program involved 10 educational modules, goal setting, and home-based physical activities monitored using an activity tracker. We assessed objective physical activity levels and behaviors using cluster analysis, aerobic fitness, and HRQoL at baseline and after the intervention (week 12). Parents were trained to reassess aerobic fitness at home at follow-up (week 24).

**Results:**

In total, 30 participants opted in, of whom 27 (90%) completed baseline assessments, and 23 (77%) commenced iBounce. Our opt-in rate was 59% (30/51), and most (19/23, 83%) of the survivors completed the intervention. More than half (13/23, 57%) of the survivors completed all 10 modules (median 10, IQR 4-10). We achieved a high retention rate (19/27, 70%) and activity tracker compliance (15/19, 79%), and there were no intervention-related adverse events. Survivors reported high satisfaction with iBounce (median enjoyment score 75%; ease-of-use score 86%), but lower satisfaction with the activity tracker (median enjoyment score 60%). Parents reported the program activities to be acceptable (median score 70%), and their overall satisfaction was 60%, potentially because of technological difficulties that resulted in the program becoming disjointed. We did not observe any significant changes in physical activity levels or HRQoL at week 12. Our subgroup analysis for changes in physical activity behaviors in participants (n=11) revealed five cluster groups: *most active*, *active*, *moderately active*, *occasionally active*, and *least active.* Of these 11 survivors, 3 (27%) moved to a more active cluster group, highlighting their engagement in more frequent and sustained bouts of moderate-to-vigorous physical activity; 6 (56%) stayed in the same cluster; and 2 (18%) moved to a less active cluster. The survivors’ mean aerobic fitness percentiles increased after completing iBounce (change +17, 95% CI 1.7-32.1; *P*=.03) but not at follow-up (*P*=.39).

**Conclusions:**

We demonstrated iBounce to be feasible for delivery and acceptable among survivors, despite some technical difficulties. The distance-delivered format provides an opportunity to engage survivors in physical activity at home and may address barriers to care, particularly for regional or remote families. We will use these pilot findings to evaluate an updated version of iBounce.

**Trial Registration:**

Australian New Zealand Clinical Trials Registry ACTRN12621000259842; https://anzctr.org.au/Trial/Registration/TrialReview.aspx?ACTRN=ACTRN12621000259842

## Introduction

### Background

Advances in childhood cancer treatments have led to significant improvements in survival rates globally [1,2]. Despite improvements in survival rates, many childhood cancer survivors (henceforth called *survivors*) are at increased risk of developing late effects and chronic diseases such as cardiovascular disease, metabolic syndrome, and obesity [3,4]. Health behavior interventions, including physical activity promotion and engagement, are crucial for preventing or minimizing the impact of these late effects [5,6]. In addition, physical activity may have a positive influence on survivors’ health-related quality of life (HRQoL) [7]. Nevertheless, many survivors do not engage in sufficient levels of physical activity [8], have poor perceptions of their activity levels [9], and have below-average fitness levels [10].

When surveyed, 60% of the survivors expressed a need for age-appropriate exercise information, and 79% reported a desire for exercise guidance [11]. A major burden for many survivors and families, especially for the 45% of this population living in rural or regional areas in Australia, is the tyranny of distance, compounded by the financial burden of travel and accommodation [12]. As a result, families from rural and regional areas have less access to supportive care, experience greater cancer-related financial hardship in survivorship than metropolitan families, and are therefore at highest risk of poor health outcomes [13,14]. Consequently, the delivery of health behavior interventions using distance-delivered technologies is a growing field to address physical inactivity and low fitness levels, as well as to improve access to services among this population [15-17].

### Objectives

This study aimed to pilot *iBounce*, a distance-delivered health education intervention for fostering health behaviors (ie, physical activity to improve fitness levels) among survivors of childhood cancer. iBounce uses a slightly modified version of iEngage (BePatient), an evidence-based health education program that provides children without chronic disease with health knowledge and practical skills to improve their physical activity behaviors and strive toward achieving recommended moderate-to-vigorous physical activity (MVPA) levels. iEngage uses a digital app on a tablet connected to wearable activity trackers that record physical activity continuously and support experiential learning [18,19]. We adapted iEngage to create the home-based iBounce intervention by modifying physical activities, educational content, and readability of the program, as well as incorporating direct parent involvement in the intervention and creating a messaging platform for participants to contact the study coordinator for technical assistance or general inquiries.

Our primary aim was to evaluate the feasibility and acceptability to survivors of using iBounce at home. Our secondary aims were to assess the impact of the iBounce intervention on survivors’ physical activity levels and behaviors, aerobic fitness, and HRQoL.

## Methods

### Participants

Our eligibility criteria included participants who (1) were aged 8 to 13 years, (2) had been diagnosed with childhood cancer, (3) were at least 12 months post cancer treatment completion or undergoing maintenance chemotherapy, (4) were able to communicate in English, and (5) had internet access at home. Participants were excluded if they (1) had cancer relapse after recruitment, (2) had a medical condition that would prohibit exercise, (3) were participating in another research study that would affect this study’s primary and secondary outcomes, or (4) had previously completed a research study <4 weeks prior. We recruited participants from Sydney Children’s Hospital in Randwick, New South Wales, Australia, between May 2019 and May 2021. Study recruitment was affected from March 2020 to May 2021 because of the COVID-19 pandemic, limiting face-to-face consultations and reducing opportunities for face-to-face communication with families.

### Ethical Considerations

Nursing staff identified eligible participants through hospital clinic lists with approval from treating oncologists. The study coordinator (LH) telephoned eligible parents or carers of survivors (henceforth called *parents*) to discuss the study, with the study invitation package sent through email or post. An initial consultation was organized with consenting parents and survivors, and study equipment was provided for participants. After written informed consent was provided by parents, survivors were enrolled in the study and provided with account log-in details for the program. The study received ethics approval through the Sydney Children’s Hospital Network Human Research Ethics Committee (HREC/18/SCHN/471), and we retrospectively registered the intervention on the Australian New Zealand Clinical Trials Registry (ANZCTN12621000259842).

### Intervention

The iBounce program uses iEngage [18-20], which includes a digital app on a tablet connected to wearable activity trackers that record physical activity continuously and support experiential learning. The iEngage digital app includes animated animal characters who guide the child through 10 self-paced educational modules that focus on different health topics, including physical activity, muscular strength, sedentary behaviors, and fitness (refer to Multimedia Appendix 1 [19] for a summary of the module topics). There is also a focus on health literacy throughout the program, which involves teaching participants to define and classify physical activity intensities (light, moderate, and vigorous), self-ratings of perceived effort during exercise, recommended guidelines relating to physical activity, sedentary behaviors, fitness, well-being (physical, mental, and social), screen-based behaviors, and sugar intake. iEngage was built for schoolchildren aged 10 to 12 years without a chronic disease; it was piloted in a rural school in New Caledonia [18] and trialed in 2 primary schools in Sydney, New South Wales, Australia, in 2017 and 2018 [19,21]. Caillaud et al [19] and Diaz et al [21] have detailed the iEngage program.

For this study, we adapted iEngage by (1) modifying the activities so that they were suitable for completion in a home setting, (2) modifying the module contents to encourage participation with family and friends, (3) expanding readability to ensure that younger children could understand the content, (4) including a messaging platform for participants to contact the study coordinator for technical assistance or general inquiries, and (5) educating parents on how to assess and administer an aerobic fitness test for their child. We aimed to target children aged 8 to 13 years because this is a critical life period for a child, when they are learning habits and becoming more independent [22]. We recommended to the participants that they complete the modules once or twice per week and synchronize their activity tracker to the program when completing each module.

### Outcome Measures

#### Overview

Survivors and parents completed questionnaires at baseline and after the intervention (week 12). We assessed survivors’ objective physical activity levels and aerobic fitness assessments at baseline and after completing the iBounce intervention. Aerobic fitness was assessed by parents at home at follow-up (week 24).

#### Feasibility

We calculated (1) opt-in rates (percentage of participants who opted into the study), (2) retention rates (percentage of participants who completed the intervention), (3) proportion of program modules completed (10 in total), and (4) activity tracker adherence (number of modules for which activity trackers were synchronized with the app) to determine feasibility. On the basis of previous similar childhood cancer physical activity interventions [8,23], we decided on opt-in and retention rates of 70% as our feasibility targets. In addition, we evaluated the feasibility of the intervention by assessing adverse events, defined as any detrimental health- or medical-related event that occurred during, or as a direct result of, exercise. Participants self-reported adverse events, which was based on the Common Terminology Criteria for Adverse Events (version 5.0) guideline [24]. The study coordinator (LH) also noted any technical difficulties experienced by participants and how they were resolved.

#### Acceptability

At 12 weeks after the intervention, the survivors completed an acceptability questionnaire that included items adapted from the Youth Satisfaction Questionnaire [25] in addition to 4 purposely designed items to measure survivors’ enjoyment and satisfaction with the iBounce program using a scale ranging from 0 to 100 (0=not at all, 100=enjoyed lots), as well as open-text fields to assess reasons for their satisfaction rating. Other acceptability questions included the following:

- Would you encourage other children to be a part of the iBounce study?
- Would you be happy to keep using the activity tracker in the future?
- Did you learn anything from using the program?

The survivors could respond *Yes*, *Unsure*, or *No* and provide reasons in open-text responses. We also asked the survivors to report on what they thought could be improved using an open-text field. In addition, we assessed parent-reported acceptability of the iBounce program using questions that were identical to those answered by the survivors and 2 additional items to assess whether their child’s participation in the iBounce study was beneficial or burdensome to them, using a 5-point Likert scale (1=*not at all*, 5=*very much so*). We decided on a rate of 70% as our acceptability target, similarly reported in previous childhood cancer physical activity interventions [8,23].

#### Physical Activity

We objectively assessed physical activity levels using the GENEActiv accelerometer (Activinsights) at baseline and after the intervention [26]. We instructed participants to wear the accelerometer on their nondominant hand for 7 consecutive days, including at night. The GENEActiv accelerometer is a research-grade waterproof wrist accelerometer that records continuous daily activity and captures acceleration along three axes (x, y, and z) with a sample frequency of 60 Hz. It shows good validity and accuracy at both wrist locations (right: *r*=0.90, left: *r*=0.91) [27]. After 7 consecutive days of wear time, participants returned the accelerometer in a prepaid-postage envelope. Once the study coordinator (LH) received the tracker, the raw data were downloaded to a computer, generating 1 data set per survivor.

To ensure that the accelerometer data were comparable for each participant, we excluded days with missing data or noncompliance. Our exclusion criteria for noncompliance were guided by the protocol described by Mattocks et al [28]: (1) an *invalid* day had <10 hours of data, (2) *nonwear* time within 1 day contained >80% of sedentary behavior from 6 AM to 9 PM (excluding sleep time), and (3) *number of valid days* was <3 days. Possible valid reasons for which data were missing include children taking the accelerometer off for certain sports that do not allow watches to be worn (eg, netball).

#### Aerobic Fitness

We assessed the survivors’ aerobic fitness using the 6-minute walk test (6MWT) at baseline, after the intervention (week 12), and at follow-up (week 24). The 6MWT has previously been used in childhood cancer survivors and is a good predictor of aerobic fitness [9,10]. Before and after the intervention, the 6MWT was administered by an accredited exercise physiologist (LH), in accordance with the American Thoracic Society recommendations [29]. During the assessment, the accredited exercise physiologist educated and demonstrated to the parent how to administer the test. Participants were encouraged to walk as fast as they could without running within 6 minutes around a 30-m track. We provided a 30-m–long rope and cones for participants to distinguish a 30-m track. Participants were instructed to walk around the rope, aiming for as many laps as they could without running. After the test, participants rated the intensity of the test using the Borg Rating of Perceived Exertion scale, which runs from 0 to 10 (0=rest, 10=maximal effort) [30]. At the week-24 follow-up period, the survivors were assessed on the 6MWT again by their parent at home using written instructions and equipment that was provided to them in their study equipment pack.

#### HRQoL Assessment

We used the EQ-5D Youth 5-Level Questionnaire (EQ-5D-Y-5L) to assess self-reported HRQoL in the survivors at baseline and 12 weeks after the intervention [31]. The questionnaire uses appropriate and child-friendly wording and comprises five dimensions—(1) mobility; (2) looking after myself; (3) doing usual activities; (4) having pain or discomfort; and (5) feeling worried, sad, or unhappy—answered on a 5-level Likert scale ranging from 1=*no problems* to 5=*I am unable to*. The EuroQoL Group’s visual analog scale accompanies the EQ-5D-Y-5L, where participants self-report their health on a scale ranging from 0 to 100 (0=*the worst health you can imagine*, 100=*the best health you can imagine*). The EQ-5D-Y-5L has been validated in children aged 8 to 16 years with pediatric conditions, including childhood cancer, with a test-retest reliability of 0.84 [32].

### Statistical Analyses

This pilot study primarily aimed to assess feasibility and acceptability; therefore, there was no initial power analysis to calculate a required sample size [8]. We aimed to enroll a sample size of up to 30 participants, which was considered sufficient for the purpose of providing initial feedback data to improve the intervention, testing the planned recruitment method, and assessing the acceptability of the intervention from the perspective of survivors and parents [33].

We analyzed data using SPSS software (version 26.0; IBM Corp) and used descriptive statistics to describe participant characteristics and evaluate the feasibility and acceptability of iBounce. We calculated the opt-in rate by dividing the total number of participants who opted into the study by the total number of invited participants (excluding participants who were unreachable or ineligible). For qualitative acceptability data, we used content analysis to interpret the meaning from the context of the text data [34]. We used the Accessibility and Remoteness Index of Australia to assess the rurality of participants’ residence according to their distance from Australian service centers [35].

This pilot was purposely not powered to evaluate the efficacy of iBounce. However, we conducted preliminary analyses to evaluate the impact of iBounce on the survivors’ physical activity levels, aerobic fitness levels, and HRQoL. On the basis of intention-to-treat principles, we used mixed effects models accounting for missing-at-random data and with participant-specific random intercepts to assess the change in (1) physical activity levels (minutes per day) and sedentary behavior (hours per day) using two time points (baseline vs after the intervention), (2) aerobic fitness levels using three time points (baseline vs after the intervention vs follow-up), and (3) HRQoL scales using two time points (baseline vs after the intervention).

We calculated means and SDs of daily time spent in sedentary behavior and MVPA using the minimum bout-filtered daily accumulated data (≥60-second bouts for sedentary behavior and ≥3-second bouts for MVPA) [36]. We compared the survivors’ daily physical activity levels with the Australian 24-Hour Movement Guidelines for children and adolescents aged 5 to 17 years [37]. The physical activity guidelines recommend that children accumulate at least 60 minutes of MVPA per day.

We performed our cluster analysis using R (RStudio), published in Open Science Framework. We processed the raw data into 1-second epoch signal vector magnitude data points of activity between 7 AM and 10 PM and then classified each second into a physical activity intensity level (sedentary, light, and MVPA) using activity cut points validated for children aged 8 to 14 years [27]. Our cluster analysis grouped participants according to the similarity of their mean daily physical activity and sedentary behaviors to characterize each cluster before and after the intervention. We used principal component analysis to maximize the variance of our accelerometer data and the elbow method for the total within-cluster sum of squares to confirm the number of acceptable clusters. All participants’ daily physical activity was clustered by eight factors: total time spent in MVPA of ≥3-second bouts and ≥30-second bouts, total time spent in sedentary behavior of ≥60-second bouts and ≥300-second bouts, frequency of ≥3-second and ≥30-second MVPA bouts, and frequency of ≥60-second and ≥300-second sedentary bouts. Informed by the studies by Diaz et al [21] and Schaefer et al [38], we selected 3-second–bout lengths to represent short MVPA bouts because activity recorded in <3 seconds typically represents agitation of the GENEActiv activity tracker or *noise*, rather than meaningful physical activity. We used bout lengths of at least 30 seconds to represent sustained MVPA. Likewise, for sedentary behavior, we used bout lengths of at least 60 seconds to represent short duration activity and bout lengths of at least 300 seconds to represent sustained activity. Next, we compared the survivors’ cluster group at baseline and after the intervention to ascertain whether they had moved to a more active cluster after the intervention, suggesting behavior change.

In addition to quantifying daily time spent in various physical activity intensities before and after the intervention, we used unsupervised data mining methods to further understand physical activity behaviors. We sought to assess behaviors defined by how the survivors’ sedentary behavior and MVPA levels were distributed throughout the day and how these levels evolved with regard to intensity, duration, and frequency of physical activity bouts. Exploring various physical activity bouts plays an important role in understanding how survivors accumulate their physical activity; for example, whether survivors engage in frequent and short bouts of MVPA or less frequent and long bouts of MVPA. To capture these various physical activity intensities, durations, frequencies, and bouts, we used the cluster analysis method proposed in earlier work [21] to analyze the impact of iBounce on the survivors’ physical activity behaviors.

For aerobic fitness, we converted the survivors’ 6MWT distance results to age- and sex-specific percentiles [39]. To assess the frequencies of self-reported HRQoL problems, we dichotomized the five levels into *no problems* (level 1) and *any problems* (levels 2, 3, 4, and 5). We analyzed the five dimensions (mobility; looking after myself; doing usual activities; having pain or discomfort; and feeling worried, sad, or unhappy) individually and determined a quality-of-life index value ranging from 0 to 1 (0=death, 1=perfect health) according to the developer’s instructions [40].

## Results

### Overview

**Figure 1:**
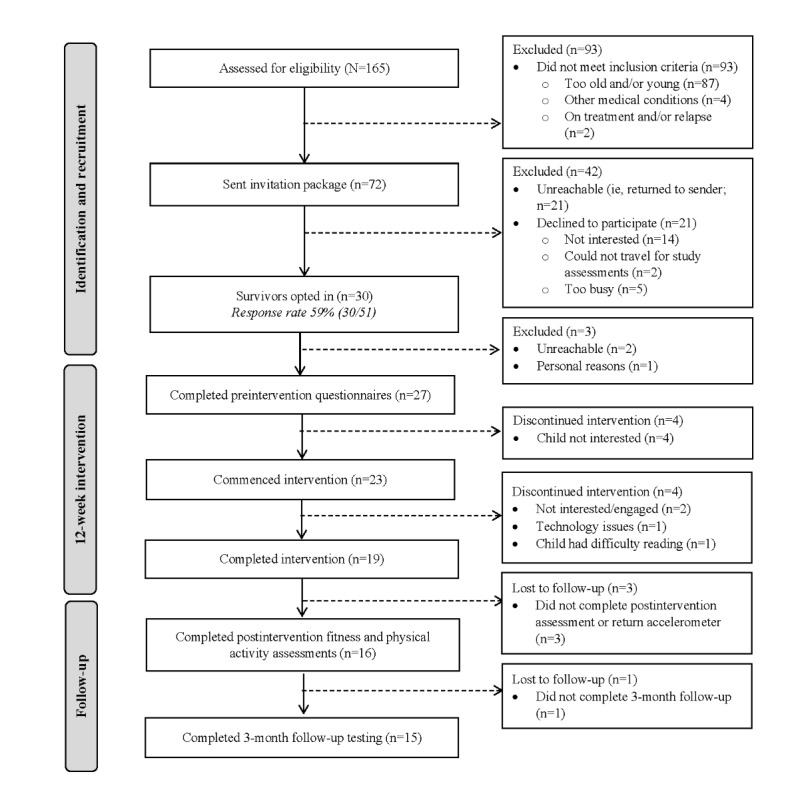
Recruitment flowchart.

Of 165 childhood cancer survivors screened for eligibility, 93 (56.4%) did not meet the inclusion criteria (refer to Figure 1 for details). We invited the remaining (72/165, 43.6%) childhood cancer survivors to enroll in our study. Of these 72 survivors, 21 (29%) actively refused (n=14, 67%, were not interested; n=5, 24%, were too busy; and n=2, 9%, were unable to travel for assessments), and 21 (29%) could not be contacted (ie, there was no response after 2 telephone calls and a voicemail). Thus, of the 72 survivors invited to enroll in our study, 30 (42%) opted into the study. The mean age of the participants was 10.2 (SD 1.5) years, 44% (12/27) were female survivors, and they were on average 5.0 (SD 3.1) years from cancer treatment completion. Participant demographic and clinical characteristics are described in Table 1.

**Table 1:** Participant baseline demographic and clinical characteristics (N=27).

Characteristics		Values
Sex (female), n (%)		12 (44)
Age during study (years), mean (SD)		10 (1.5)
Time since treatment completion (years), mean (SD)^a^		5 (3.1)
**Cancer diagnosis, n (%)**		
	Acute lymphoblastic leukemia	15 (56)
	Neuroblastoma	4 (15)
	Burkitt lymphoma	3 (11)
	Other malignancies^b^	5 (18)
**Treatment received, n (%)^c^**		
	Chemotherapy	26 (96)
	Surgery	17 (63)
	Radiotherapy	4 (15)
	Bone marrow transplant or stem cell transplant	4 (15)
**Rurality, n (%)^d^**		
	Major city	23 (85)
	Regional	4 (15)
**Parent education, n (%)**		
	High school	3 (11)
	Certificate, diploma, or apprenticeship	10 (37)
	University degree	10 (37)
	Postgraduate degree	4 (15)

^a^The data of 2 participants were missing.

^b^Other malignancies include hepatoblastoma, non-Hodgkin lymphoma, Wilms tumor, and germ cell tumor.

^c^Participants may have had >1 treatment.

^d^According to the Accessibility and Remoteness Index of Australia, which categorizes regions according to their accessibility to services.

### Feasibility

The opt-in rate was 59% (30/51; of the 72 participants who were sent the invitation package, 21, 29% were unreachable). Of the 30 participants who verbally opted into the study, 2 (7%) could not be contacted, and 1 (3%) decided to withdraw from the study for personal reasons; the remaining 27 (90%) participants completed baseline assessments. However, of these 27 participants, 3 (11%) withdrew after completing baseline assessments, and 1 (4%) withdrew after completing only the fitness assessment, primarily because of lack of interest. Of the 3 participants who withdrew after completing baseline assessments, 1 (33%) male participant aged 13 years expressed that he felt too old for the program. Of the 30 participants who verbally opted into the study, 23 (77%) commenced the iBounce program. Of these 23 participants, 4 (17%) withdrew after commencing iBounce because of lack of engagement (n=2, 50%), technology issues (n=1, 25%), and difficulty reading (n=1, 25%), resulting in a 70% (19/27) retention rate. Of the 23 participants who commenced iBounce, 19 (83%) completed the intervention. There were no intervention-related adverse events reported, although we recorded a nonserious adverse event where a participant fractured their arm in an incident unrelated to the intervention, and they were still able to continue.

Of the 23 participants who commenced the program, 13 (57%) completed all 10 modules (median 10, IQR 4-10). Activity tracker engagement was high, with 79% (15/19) of the participants synchronizing their activity tracker to the program for ≥7 iBounce modules. The median number of modules that activity trackers were worn for, and synchronized to, was 9 (IQR 2-10).

In total, 70% (16/23) of the survivors completed the postintervention fitness test and returned their accelerometers, and 65% (15/23) completed the week-24 follow-up assessment.

### Technical Difficulties

More than half (13/23, 57%) of the participants reported at least one technical difficulty. The most commonly reported technical difficulty was synchronization issues between the Misfit Ray (Fossil Group) activity tracker and the app (13/20, 65%). Of the 4 participants who dropped out after commencing the intervention, 2 (50%) discontinued the study because of these technical difficulties. All remaining technical issues were resolved through consultations (telephone call, SMS text messaging, or email) with the study coordinator (LH). Solutions to the technical difficulties involved replacing the activity trackers, sending new batteries to participants, and resetting the modules.

### Acceptability

#### Survivor-Reported Acceptability

**Figure 2:**
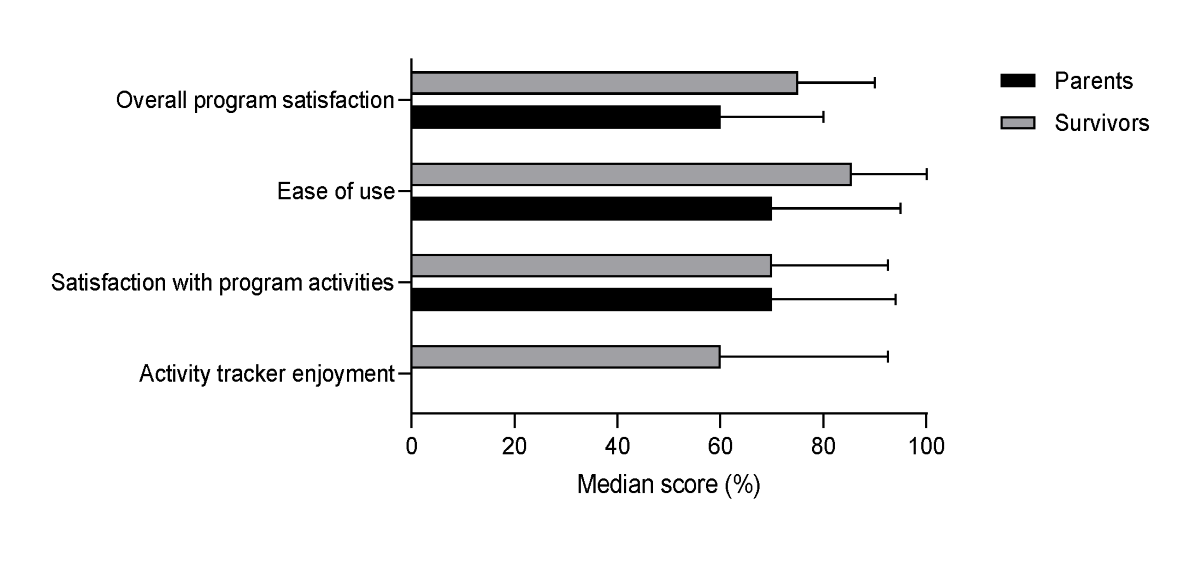
Median and range values of scores for survivor and parent satisfaction ratings of iBounce at 12 weeks after the intervention (survivors: n=14, parents: n=15).

Regarding satisfaction with iBounce, the survivors reported median scores of 86%, 75%, and 70% for ease of use, overall program satisfaction, and satisfaction with program activities, respectively (Figure 2). Qualitatively, the survivors reported that the program activities were fun, convenient, and engaging. Of the 14 survivors who responded to questionnaires, 12 (86%) also reported having learned from the program, and 9 (64%) stated that they would encourage other children to participate in the iBounce program. In total, 22% (3/14) of the survivors reported that they were unsure whether they would recommend participating in the iBounce program to other children, and 14% (2/14) reported that they would not recommend iBounce to other children because it was boring, and the activity tracker was unreliable. Reasons for recommendations included benefits of iBounce as an engaging and educational program for children, with a survivor highlighting that iBounce may be helpful for their friends; for example, “because some of my friends aren’t that healthy and this would direct them into the right track” [Male survivor aged 13 years]. Multimedia Appendix 2 summarizes survivors’ and parents’ responses to open-ended questions.

**Figure 3:**
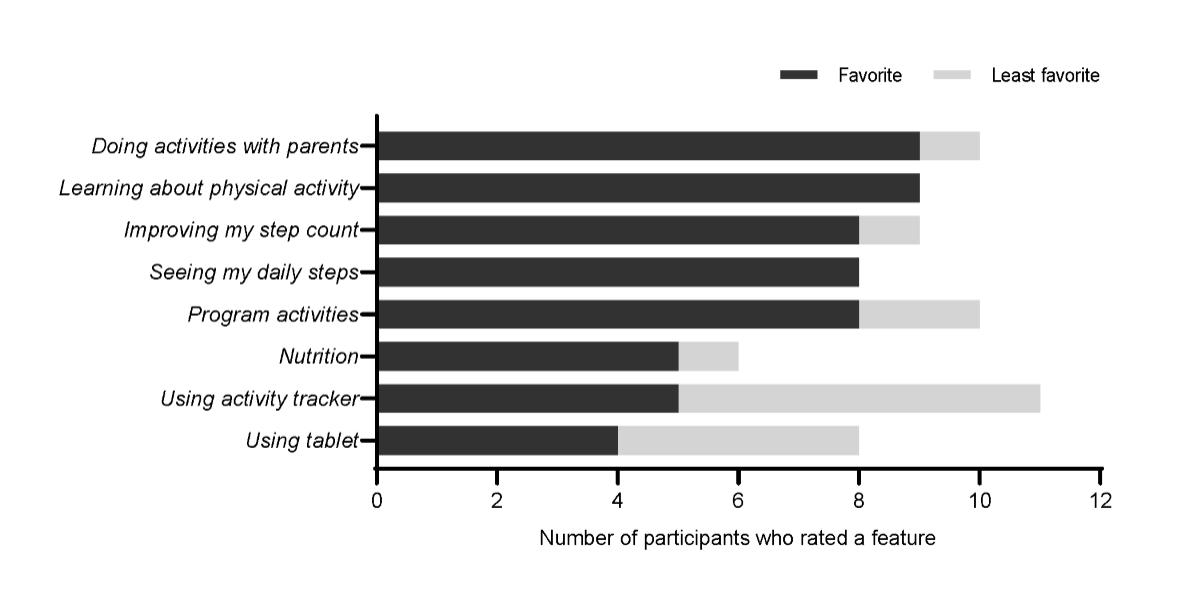
Survivors’ acceptability ratings of iBounce program features at 12 weeks after the intervention (n=14). It should be noted that participants were able to choose >1 option.

Of the 14 survivors, 9 (64%) rated completing activities with their parents and learning about physical activity as their top-rated program features (Figure 3). The bottom-rated features were using the activity tracker (7/14, 50%) and using the tablet (5/14, 36%).

The median score for activity tracker enjoyment was 60% (IQR 40%-92.5%; Figure 2). In total, 43% (6/14) of the participants enjoyed using the activity tracker, highlighting it as a motivational and educational tool; for example, a survivor reported, “It was so good to be able to see how many steps I could do, then try to improve*”* [Female survivor aged 11 years]*.* However, 57% (8/14) of the survivors reported dissatisfaction with the activity tracker because of technical difficulties such as issues with synchronization and connection.

Half of the participants (7/14, 50%) indicated that they would be happy to keep using the activity tracker in the future, whereas 14% (2/14) of the participants were unsure, and more than one-third (5/14, 36%) reported that they would not use the activity tracker in the future because of limitations such as lack of real-time feedback on the tracker and absence of time display or heart rate monitoring, with a survivor reporting, “...I normally use a tracker with a display [so] that I can check my steps and heart beating” [Female survivor aged 8 years]. The open-ended feedback we received regarding improvements to the intervention also reflected suggestions to improve the Misfit Ray activity tracker. Of the 9 participants who provided suggestions to improve iBounce, 6 (67%) provided suggestions related to changes to the activity tracker, 2 (22%) reported *unsure*, and 1 (11%) suggested changes to the program app.

#### Parent-Reported Acceptability

The median score for parents’ satisfaction with program activities was 70% (IQR 50%-95%; Figure 2). Common themes in the qualitative data included positive endorsements of iBounce as a program that engaged their child in physical activity, raised awareness of health behaviors for both survivors and parents, started a conversation about well-being, and facilitated parent involvement. The median score for satisfaction with the program was 60% (IQR 50%-80%). Parents who were not satisfied with the program (9/14, 64%, scored ≤60) reported technological difficulties such as the Misfit Ray activity tracker’s batteries needing to be replaced, which interrupted the program; the program not being targeted to their child’s age; and the program being confusing to set up.

In total, 47% (7/15) of the parents reported that participation in the study was beneficial to them (n=1, 14%, reported *very much so*, and n=6, 86%, reported *quite a bit*). One-third (5/15, 33%) reported that participation was *somewhat* beneficial. A few parents reported that participation was *a little bit* (1/15, 7%) and *not at all* (2/15, 13%) beneficial to them. The parents indicated several benefits of iBounce, including the program serving as a reminder or motivation for their child to exercise, encouraging a better attitude toward exercise, and motivating for the parent themselves to promote physical activity for their child.

Many of the parents reported that participation was *not at all* (4/15, 27%) or *a little bit* (6/15, 40%) burdensome, whereas some reported that participation was *somewhat* (3/15, 20%) or *quite a bit* (2/15, 13%) burdensome. Some (4/15, 27%) of the parents described technical difficulties such as the lack of a *back* button on the app and problems synchronizing the activity tracker. In total, 20% (3/15) of the parents reported that having to monitor or remind their child to do the program was burdensome. The majority (10/15, 67%) of parents indicated that they would recommend to other children that they participate in this study, including those who reported the study to be *not at all* (3/15, 20%), *quite a bit* (1/15, 7%), *somewhat* (2/15, 13%), and *a little bit* (4/15, 27%) burdensome. Parents who would recommend iBounce to others endorsed that iBounce highlighted the importance of health behaviors for their child after cancer treatment and that it encouraged them to engage in physical activity.

### Physical Activity

Of the 30 participants who opted into the study, 3 (10%) were excluded after opting in, 3 (10%) discontinued the intervention, and 24 (80%) returned the accelerometers at baseline. After data extraction and quality assessment, we excluded 12% (3/24) of the participants because of insufficient available accelerometer data. We therefore included data from 88% (21/24) of the participants at baseline. Of the 19 participants who completed the intervention, 16 (84%) returned their accelerometer for the postintervention assessment. Of these 16 participants, 4 (25%) had incomplete data sets, resulting in the data sets of 12 (75%) participants being available for analysis at the postintervention assessment. Overall, we analyzed 11 complete data sets for both pre- and postintervention assessments because, of these 12 participants, 1 (8%) had insufficient baseline data.

At baseline, the survivors were engaging in an average 41.7 (SD 17.7) minutes per day of MVPA (Table 2). Most (86%, 18/21) of the survivors were not meeting physical activity guidelines over a week. On average, the survivors who met the physical activity guidelines engaged in sufficient MVPA levels on mean 1.2 (SD 1.7) days per week. The survivors did not increase their mean daily MVPA from before to after the intervention (mean 39.2, SD 24.7 minutes per day, change –2.5, SE 7.4, 95% CI –17.6 to 12.6; *P*=.74; Table 2). On average, the survivors spent 5.6 (SD 1.6) hours per day in sedentary behaviors of bouts of at least 60 seconds (excluding sleep time from 10 PM to 7 AM). There was no evidence of change in sedentary behaviors between before and after the intervention (mean 5.6, SD 1.6 hours per day, change +0.02, SE 0.6, 95% CI –1.13 to 1.18; *P*=.97).

We assessed changes in physical activity behaviors among the 11 survivors who had complete accelerometer data at pre- and postintervention assessments. We retained 4 principal components, and our cluster analysis resulted in 5 acceptable cluster groups. Cluster 1 represents the most active group that meets the physical activity guidelines and engages in the most frequent bouts of MVPA, whereas cluster 5 represents the least active group with infrequent and sustained bouts of sedentary behaviors (Textbox 1). Multimedia Appendix 3 shows the duration and frequency of bout lengths in MVPA and sedentary behaviors for each cluster.

**Table 2:** Change in objectively measured physical activity levels and sedentary behaviors.

		Before the intervention (n=21)		After the intervention (n=12)		SE (95% CI)		*P* value	
MVPA^a^ (≥3-second bouts), minutes per day, mean (SD)		41.7 (17.7)		39.2 (23.7)		7.4 (–17.6 to 12.6)		.74	
Sedentary behaviors^b^ (≥60-second bouts), hours per day, mean (SD)		5.6 (1.6)		5.6 (1.6)		0.6 (–1.1 to 1.2)		.97	
Number of days meeting guidelines: mean (SD)^c^		1.2 (1.7)		1.3 (2.7)		N/A^d^		N/A	
**Number of participants meeting guidelines, n (%)^c^**									
	Did not meet guidelines (<60 minutes per day)		18 (86)		10 (83)		N/A		N/A
	Met guidelines (≥60 minutes per day)		3 (14)		2 (17)		N/A		N/A

^a^MVPA: moderate-to-vigorous physical activity.

^b^Sleep time (10 PM to 7 AM) was excluded from our analyses.

^c^Physical activity levels were compared with recommended physical activity guidelines of at least 60 minutes of moderate-to-vigorous physical activity per day for children and adolescents, including weekends.

^d^N/A: not applicable.

**Definition of each cluster group.**

**Cluster group and definition**

Cluster 1: most active

- Meets the daily physical activity recommended guidelines (≥60 minutes per day)
- Engages in the most time spent in moderate-to-vigorous physical activity (MVPA) in ≥3-second bouts that is the most frequent (637 bouts)
- Engages in frequent and sustained levels of sedentary behavior in ≥60-second bouts (>300 minutes, 77 bouts) and ≥300-second bouts (>150 minutes, 11 bouts)

Cluster 2: active

- Engages in frequent and high levels of MVPA (47.2 minutes, 495 bouts)
- Engages in the least amount of sedentary behavior in ≥60-second bouts (<200 minutes, 33 bouts) and ≥300-second bouts (<150 minutes, 6 bouts)

Cluster 3: moderately active

- Engages in the most time spent in longer bouts of MVPA (≥30-second bouts: 6 minutes, 6 bouts)
- Engages in frequent and sustained levels of sedentary behavior in ≥60-second bouts (>350 minutes, 109 bouts) and ≥300-second bouts (>190 minutes, 9 bouts)

Cluster 4: occasionally active

- Engages in occasional and low levels of MVPA in ≥3-second bouts (26 minutes, 298 bouts) and ≥30-second bouts (1 minute, 2 bouts)
- Engages in moderate levels of sedentary behavior (≥60-second bouts: >300 minutes, 111 bouts) but less sustained (≥300-second bouts: 130 minutes, 9 bouts)

Cluster 5: least active

- Engages in low levels of MVPA (≥3-second bouts: 21 minutes, 245 bouts) that is not sustained (≥30-second bouts: 2 minutes, 2 bouts)
- Engages in the most frequent and most time spent in sedentary behavior (≥60-second bouts: >500 minutes, 57 bouts) that is sustained (≥300-second bouts: >420 minutes, 13 bouts)

Table 3 is a visual representation of the movement of participants among the cluster groups before and after the intervention. In total, 27% (3/11) of the participants moved to a more active cluster group after completing the iBounce intervention, highlighting an increase in more frequent and sustained bouts of MVPA, whereas 55% (6/11) stayed in the same cluster; however, of these 6 participants, 2 (33%) were already highly active at baseline. Of the 11 participants, 2 (18%) moved to a less active cluster after completing the iBounce intervention.

**Table 3:** Daily cluster movement matrix.

		To daily cluster (after the intervention)				
		1	2	3	4	5
**From daily cluster (before the intervention)**						
	1	1^a^	N/A^b^	N/A	N/A	N/A
	2	N/A	1^a^	N/A	N/A	N/A
	3	1^c^	N/A	1^a^	1^d^	N/A
	4	N/A	1^c^	N/A	3^a^	1^d^
	5	N/A	N/A	N/A	1^c^	N/A

^a^The diagonal area indicates no improvement in physical activity levels (stayed in the same cluster).

^b^N/A: not applicable.

^c^Indicates desirable movements (moving to a more active cluster group after completing iBounce).

^d^Indicates unfavorable movements (moving to a less active cluster group after completing iBounce).

### Aerobic Fitness

**Figure 4:**
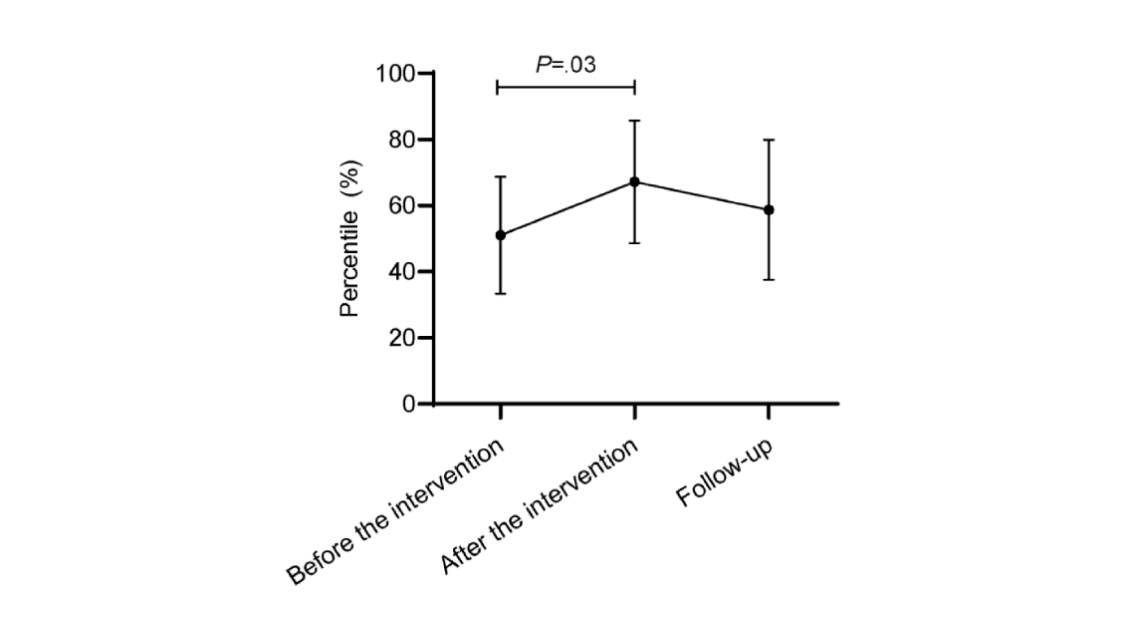
Change in aerobic fitness percentile means from before the intervention and after the intervention to week-24 follow-up.

At baseline, the survivors’ mean aerobic fitness performance was at the 44th (SD 32) percentile, indicating average fitness levels. The survivors’ aerobic fitness increased from before to after the intervention (mean 61, SD 36 percentile, change +17, 95% CI 1.7-32.1; *P*=.03). There was no difference between the postintervention (week 12) and follow-up assessments at week 24 (mean 54, SD 38 percentile, change –7, 95% CI –23.3 to 9.4; *P*=.39; Figure 4). In addition, there was no change between baseline and follow-up aerobic fitness (change +10, 95% CI –6.9 to 26.8; *P*=.23).

### HRQoL Scores

**Figure 5:**
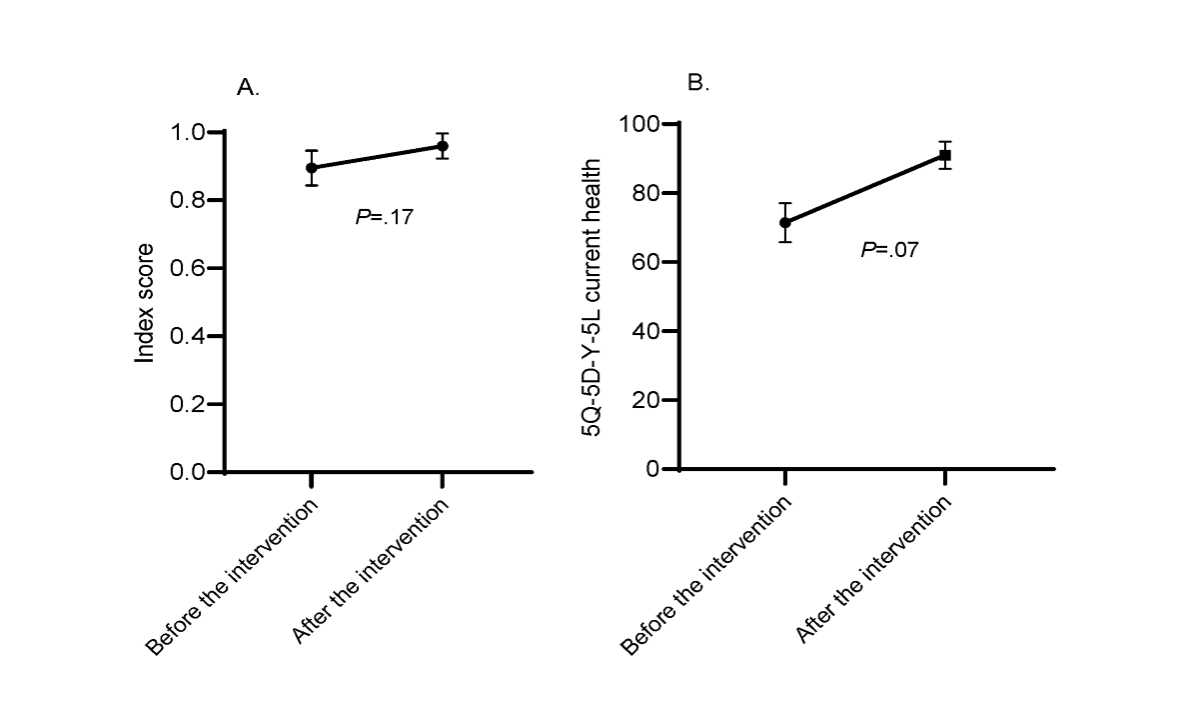
(A) Change in the EQ-5D Youth 5-Level Questionnaire (EQ-5D-Y-5L) health-related quality-of-life mean index scores and (B) mean current health ratings between preintervention and postintervention assessments. The index scores range from 0 to 1 (0=death, 1=perfect health), and the current health ratings range from 0 to 100 (0=worst health, 100=best health).

The survivors commonly indicated problems relating to pain or discomfort (14/26, 54%), anxiety or depression (9/26, 35%), activities of daily living (7/26, 27%), and mobility (7/26, 27%) at baseline (Multimedia Appendix 4). The survivors’ average HRQoL score at baseline was 0.89 (SD 0.13; range 0.47-1.00), with 42% (11/26) of the survivors reporting perfect health (score=1.00). There was no significant change in the EQ-5D-Y-5L index score from before to after the intervention (mean 0.89, SD 0.04, change +0.07, SE 0.05, 95% CI –0.03 to 0.16; *P*=.17; Figure 5A). On average, the survivors reported their overall health at baseline as 71.5 out of 100 (SD 28.8; range 0-100). There was insufficient evidence to indicate that the survivors’ overall health improved from before to after the intervention (mean 91.0, SD 11.2, change +19.5, SE 10.5, 95% CI –1.89 to 40.96; *P*=.07; Figure 5B).

## Discussion

### Principal Findings

Distance-delivered physical activity interventions are promising and feasible programs that can offer support and engagement among survivors and families in improving health behaviors [16]. The iBounce program seems to be safe, feasible, and acceptable, demonstrating moderate opt-in and high retention rates, reflecting that families were interested in physical activity support in survivorship. Our opt-in rate was lower than our feasibility target because of the challenge of study recruitment and potentially the negative impacts of the COVID-19 pandemic. In total, 56.4% (93/165) of the potential participants who were screened did not meet the eligibility criteria, and 58% (42/72) of the eligible participants either declined participation or were unreachable. The challenge of participant recruitment for health behavior interventions among children diagnosed with cancer is common [8,41]. However, our challenges may have been exacerbated because of recruitment from a single site and parents not wanting to commit to research interventions because of the unknown risk of COVID-19 for unvaccinated children diagnosed with cancer and their immunocompromised status [42]. Despite the lower-than-anticipated opt-in rate, the retention rate and program engagement were high, demonstrating that once the survivors expressed interest in the iBounce program, they were highly receptive to it. Activity tracker compliance was also lower than the feasibility target that we set, potentially because of the survivors’ preference for a monitor that showed their activity (eg, steps achieved) [43] and the technical difficulties relating to synchronization, which may have affected adherence. Some qualitative comments by the survivors further reflected their frustration with the activity tracker (Multimedia Appendix 2). Future research should explore survivors’ priorities with regard to using wearable activity trackers to optimize their acceptability and optimal use. Parent-reported acceptability of the program was also lower than anticipated, potentially because of the technological difficulties that resulted in the program becoming disjointed. One-third of the parents reported iBounce to be *somewhat* (3/15, 20%) or *quite a bit* (2/15, 13%) burdensome, and their qualitative comments were valuable, highlighting the technical difficulties, their dissatisfaction with the Misfit Ray activity tracker, and the suggestion that iBounce may be better suited to younger children. Despite the technical difficulties experienced by participants, it is encouraging that these issues did not seem to adversely affect participant engagement or the delivery of the program content. Future trials of iBounce should include a *troubleshooting* pamphlet with common technical issues and solutions to improve participant retention and reduce burden for the study coordinator.

Most (12/14, 86%) of the survivors were satisfied with the program, particularly the activities that involved their family and friends and content that facilitated health behavior education. Parents also reported high satisfaction with the program activities that encouraged time with their child. Family and peer involvement was a vital element of iBounce, where the survivors were encouraged to exercise, and share their knowledge and skills, with family and friends [19]. We also directly involved parents in the program to assist in the assessment of aerobic fitness at follow-up. There is some evidence to support that lifestyle interventions that include direct parent involvement have demonstrated positive outcomes among childhood cancer survivors [44,45]. A home-based exercise study for children aged <18 years on treatment for acute lymphoblastic leukemia engaged parents through involving them in attending and supervising exercise sessions with their children [46]. The researchers found an improvement in participants’ physical activity stage of change after the 6-week intervention [46]. Tanir and Kuguoglu [47] also involved parents in a hospital- and home-based exercise intervention for survivors of acute lymphoblastic leukemia aged 8 to 11 years by requiring at least one parent to attend the exercise sessions with their child for support and motivation. Participants in the study showed significant improvements in physical fitness and muscular strength [47]. Likewise, participants in our study improved their aerobic fitness after 12 weeks. Such findings highlight the potential benefit of involving parents in lifestyle interventions because they may be an avenue of support as well as for promoting behavior change in survivors, particularly at home.

We observed a significant increase in aerobic fitness from before to after the intervention; however, this change was not sustained at the week-24 follow-up. It may be likely that survivors need more support to maintain their behavior changes over time, such as the inclusion of booster sessions to reinforce health behavior messages. Booster sessions have previously been shown to increase long-term impacts [48]. In addition, it is possible that the reliability and accuracy of the results may have been affected because the 6MWT was administered at home, supervised by the parent instead of a qualified exercise professional. Tests of physical functioning and aerobic capacity are commonly used in clinical practice and research for identifying fitness levels and evaluating the efficacy of exercise programs [10,49]. However, the need to travel to a supervised clinic or hospital for assessments is a recognized barrier to exercise participation and a burden, especially for those living in rural and remote regions [50]. Future studies should validate or develop simple, home-based functional assessments for researchers or clinicians to facilitate distance-based exercise testing for childhood cancer survivors. The development of accurate and reliable home-based functional assessments has the potential to enable support and reduce the burden for survivors and the health care system [51].

Despite the encouraging feasibility and acceptability data, our early data did not suggest any significant improvements in the survivors’ physical activity levels after participating in the iBounce program. The lack of change in time spent in MVPA among childhood cancer survivors is consistent with previous distance-delivered exercise interventions [23,52,53]. It is possible that iBounce may not be effective in improving physical activity levels. Our small sample size may also have resulted in an insufficient number of participants to demonstrate an effect, although the primary aim of our pilot study was to assess feasibility. However, a strength of our study was our novel investigation of physical activity behaviors using cluster analysis. To complement our assessment of time spent in MVPA before and after the intervention, we used the richness of the accelerometer data and explored the continuity and duration of various physical activity bouts. Physical activity bouts (eg, frequent short bouts or infrequent long bouts) are important to understand how participants achieve their physical activity levels and how they are distributed over the day or week [21,54]. Our cluster analysis showed that 27% (3/11) of the participants moved to a more active cluster, indicating that they increased the frequency and duration of MVPA bouts. Although the total time they spent in MVPA may not have changed after the intervention, the length of bouts increased, which might suggest more structured activity. In total, 18% (2/11) of the participants moved to a less active cluster; however, of these 2 participants, 1 (50%) decreased their sedentary behavior, still indicating a positive outcome. These changes in patterns are useful in understanding the impact of iBounce on survivors’ physical activity and sedentary behaviors. Future studies should aim to examine the impact of interventions on various patterns of physical activity, including bouts, frequencies, and intensities.

Improvements in HRQoL after physical activity have previously been reported in childhood cancer survivors [55-57]. Physical activity may improve or maintain aspects of HRQoL, including physical and cognitive function, and reduce cancer-related worry [58]. The HRQoL measures in our study were not statistically different from baseline to 12 weeks after the intervention, potentially because of our small sample size. Compared with normative data in noncancer populations, iBounce participants had mean scores that were similar to those of their noncancer peers, demonstrating high quality of life to begin with [31].

### Limitations

The iBounce intervention used an adapted version of the iEngage program, which had already gone through rigorous pilot testing among primary school–aged children [18,19]. The distance-delivered format of our study enabled the survivors to access iBounce and engage in health behaviors, even throughout the COVID-19 pandemic, highlighting the relevance and applicability of iBounce. Although iBounce was available for eligible participants throughout the pandemic, government stay-at-home health orders and social distancing practices limited face-to-face consultations and communication with families, which affected some pre- and postintervention assessments such as the 6MWT. At the week-24 follow-up, parents assessed their child using the 6MWT, a method that has not yet been validated for parents to administer at home. However, the simplicity, low cost, and minimal requirement of equipment for the 6MWT allowed us to educate survivors and families on how to easily assess fitness at home. Another limitation of this study was the ad hoc monitoring of adverse events, which may have led to underreporting of events, particularly lower-grade or nonserious adverse events. Although symptoms such as muscle soreness or fatigue reflect common responses to exercise, these low-grade adverse events are important to report to provide confidence that unsupervised or home-based exercise is safe after cancer treatment [59]. Therefore, future distance-delivered physical activity interventions should regularly monitor for adverse events using a standardized approach throughout the intervention to improve adverse-event reporting and intervention quality. Our sample was heterogeneous in terms of cancer diagnoses and included participants who had average fitness levels at baseline, which may overrepresent survivors who were more active, potentially biasing the results. Furthermore, most (23/27, 85%) of the participants in our study were living in metropolitan areas, and families from regional or rural regions were underrepresented [12]. Potential reasons for the low opt-in rate among rural survivors may be due to the fact that fewer rural families were invited to the study as a result of our single recruitment site, which captures a smaller portion of rural families within New South Wales. Our distance-delivered intervention may likely benefit survivors from regional or rural areas; yet, additional recruitment strategies are needed to engage rural survivors to maximize successful adoption. Our sample also included highly educated parents, and there was no representation from brain tumor survivors. It is not clear why no families of children with a central nervous system (CNS) tumor participated in our study. It may be possible that the program was less appealing to patients with a CNS tumor because of their burden of long-term side effects such as cognitive difficulties, fatigue, and balance difficulties [60]. Future research could explore the needs of patients with a CNS tumor in more depth and consider developing a more tailored intervention for this group. Our intervention focused on English-speaking participants; future trials of iBounce should consider collaborating with non–English-speaking and culturally and linguistically diverse populations because of their increased barriers to accessing care and poorer health outcomes [61].

On the basis of our pilot feasibility and acceptability data, planned changes to iBounce will include ongoing collaboration with childhood cancer survivors to identify their preferred type of wearable activity tracker that will assist them in maintaining or improving their physical activity levels. Further collaborative efforts such as using co-design methods with survivors and parents will be used to update and improve the iBounce design and educational module content to suit a broader range of survivors, including older children and adolescents. To address the technical difficulties experienced by survivors and parents, we plan to streamline the app and remove the need for participants to synchronize the tracker to the program, which was often the cause of the technical issues. We will also provide a troubleshooting resource to reduce burden on participants needing to contact the study coordinator for assistance.

### Conclusions

Our digital health education program, iBounce, proved to be feasible and acceptable among childhood cancer survivors. We experienced some recruitment challenges and technical difficulties that resulted in an opt-in rate and module completion rate that was lower than the feasibility targets that we had set. Despite these challenges, after participants did opt in to the program, we found that it was feasible to deliver iBounce and that survivors were highly engaged and enjoyed participating in home-based physical activities with their family. Our preliminary efficacy results are promising, highlighting the potential of iBounce to improve survivors’ fitness levels after completing the program. Our positive feasibility and acceptability data warrant further investigation in a well-powered trial that also addresses the technical issues experienced in this pilot study. The digital aspect of iBounce has the potential to educate, engage, and reach a high proportion of survivors and their families with regard to positive health behaviors at home, no matter where they reside.

## Appendix

Multimedia Appendix 1 Module topics adapted from iEngage.

Multimedia Appendix 2 Survivor and parent responses to open-ended questions about the acceptability of iBounce.

Multimedia Appendix 3 Daily clusters.

Multimedia Appendix 4 Frequencies and proportions of reported health-related quality of life problems from the dichotomized EQ-5D Youth 5-Level Questionnaire.

Multimedia Appendix 5 CONSORT-EHEALTH checklist (V 1.6.1).

## Abbreviations

6MWT: 6-minute walk test
CNS: central nervous system
EQ-5D-Y-5L: EQ-5D Youth 5-Level Questionnaire
HRQoL: health-related quality of life
MVPA: moderate-to-vigorous physical activity
