# Supplementary material for: A Digital Educational Intervention With Wearable Activity Trackers to Support Health Behaviors Among Childhood Cancer Survivors: Pilot Feasibility and Acceptability Study
Source: JMIR Cancer. 2022 Aug 17;8(3):e38367. doi: 10.2196/38367 (PMC9434388; doi:10.2196/38367)
Supplement: Multimedia Appendix 1 [file cancer_v8i3e38367_app1.docx]

**Multimedia Appendix 1. Module topics adapted from iEngage© (Caillaud et al. 2022)**

| **Module** | **Topic** |
| --- | --- |
| 1 | Introduction to the iBounce program |
| 2 | Physical activity |
| 3 | Sedentary behaviours |
| 4 | Health and sedentary behaviours |
| 5 | Health and physical activity |
| 6 | Intensity of physical activity, rate of perceived exertion |
| 7 | Physical fitness |
| 8 | Muscle strength, power and flexibility |
| 9 | Sugar intake |
| 10 | Summary module: Move more and eat better |
