# Supplementary material for: A Digital Educational Intervention With Wearable Activity Trackers to Support Health Behaviors Among Childhood Cancer Survivors: Pilot Feasibility and Acceptability Study
Source: JMIR Cancer. 2022 Aug 17;8(3):e38367. doi: 10.2196/38367 (PMC9434388; doi:10.2196/38367)
Supplement: Multimedia Appendix 2 [file cancer_v8i3e38367_app2.docx]

**Multimedia Appendix 2. Survivor and parent responses to open-ended questions about the acceptability of iBounce.**

| **Survivor themes** |
| --- |
| **Satisfaction with iBounce** |
| - *It promotes good health and what I can do to improve my wellbeing* - *I liked how they taught me a lot about health.* - *Was good learning about healthy ways* - *I learned about sugar and how much you should have per day and much more* - *To not eat that much sugar because it can have a negative impact* - *That its important to look after your body* - *Helps people understand what really happens in the world of fitness and helps them become more aware of what they do and dont do* |
| **Perceived benefits – innovative, enjoyable, convenient and attractive design** |
| - *Was something different* - *You could do anytime like when you have free time* - *Because everyone could do them and they are fun* - *Because it had some cool pictures and I think the little kids will like them if this goes on in the* - *Because there were some new exercises that I have never done* - *It is fun, but hard work. Sometimes i missed a day and could not achieve my goals* - *It is fun and i had learned a lot* - *Because ot is really fun and also learn a lot about health* - *It was fun to use a tracker and i was able to see my fitness levels* - *It was encouraging on my health* - *it made me move* - *It made me want to be more active* - *It was so good to be able to see how many steps i could do then try to improve* |
| **Perceived benefits – motivation** |
| - *It was encouraging on my health* - *it made me move* - *It made me want to be more active* - *It was so good to be able to see how many steps i could do then try to improve* |
| **Perceived benefits – learnings** |
| - *I learned that I need at least 60 min of moderate exercise per day* - *I learned that you should do 12000 steps and 60 mins of moderate or vigorous activity every day* - *you should exercise each day to keep healthy* - *It's good to do exercise everyday and I think it's easy to forget how important it is* |
| **Perceived benefits – using an activity tracker** |
| - *It was cool to see how many steps I did* - *Got to see how many steps I did* - *Enjoyed wearing it and got to keep it* - *I liked being able to see how many steps I did* - *It was comfortable* - *I can keep track of my steps* - *Because it was comfortable and light.* |
| **Reasons for recommending peers to do iBounce** |
| - *People might not be ommitted but they also might be it depends what type of person you are* - *Makes them exercise* - *Incourge them to become healthy* - *it’s good for your mental health*   *Because some of my friends aren’t that healthy and this would direct them into the rigot track* |
| **Reasons for dissatisfaction with the activity tracker** |
| - *Tracker was unreliable* - *Because you have to log in everyday and if you don't it stops connecting* - *i did not like wearing it, not useful i want something i can see time on* - *Because i normally use a tracker with a display that i can check my steps and heart beating* - *I did not like the activity tracker because there was a lot of issues and one was that it couldn't connect* - *I already have a tracker and had to use one in each arm. Sometimes the tracker did not show my effort.* |
| **Suggestions to improve the activity tracker** |
| - *To be able to be more flexible to record various types of activities e.g bike riding and skateboarding* - *The tracker could have a screen so we could see the results before we have to download.* - *i couldn't see my results or the time on it which would have been better* |
| **Parent themes** |
| **Satisfaction with iBounce** |
| - *Got him moving which was great* - *It made [child] out of comfort zone and move him* - *Forced him to keep moving so got him away from electronics* - *It did get him moving a little more than usual.* - *Was just good for him to be exercising* - *The tracking bracelet was a good tool to encourage [child] to be enthusiastic about tracking her day. It also helps that the bracelet looks cool* |
| **Perceived benefits – raised awareness of health behaviours** |
| - *Facilitated awareness of good nutrition and physical activity* - *We could do it at home with support from Lauren via phone/text or email. It also forced [child] to think about exercise choices and benefits of exercise.* - *It's a great idea, makes you realise how much fitness they lose.* - *That it tries to get kids linking up exercise with wellbeing* - *Served as a reminder of the importance of exercise* - *I did notice a better attitude towards exercise* |
| **Perceived benefits – supported health behaviour education** |
| - *It encourages the child to take ownership of exercise after treatment* - *Keeps them moving and understanding the importance of it* - *To make them understand healthy lifestyle* - *The information provided to my child about creating healthy habits was valuable.* |
| **Perceived benefits – started conversations about health behaviours** |
| - *The program got us to talk about [child] activities and got him thinking in a more reflective way about his food habits.* - *Got us talking about healthy lifestyles* - *Got conversations going* |
| **Perceived benefits – facilitated parent involvement** |
| - *We started to exercise together but that has fallen by the wayside now. It was good while it lasted though.* - *It was good to exercise with her* - *It was beneficial in motivating me to get my child up and moving even though he didn't want to* |
| **Perceived burdens** |
| - *I sometimes had to ask him a few times to start the exercise for that day* - *[child] didn't like it so i had to badger him* - *Unfortunately, my child was very difficult to motivate to complete the program as he has been sedentary for so long.* |
| **Suggestions: iBounce may be better suited to younger survivors** |
| - *It isn't really targeted at our age group (12 years)* - *The IT needs to be streamlined to make the barriers/exercises disappear. The app is targeted to younger audiences ([child] is 12 and it was too young for her)* - *Probably suited to younger kids and it has a targeted approach* |
| **Suggestions: to include more exercises, to increase the difficulty of challenges or to include parents in a competitive way** |
| - *Incorporate more activities that they need to complete and take ownership for. For example, they need to set up an obstacle/fitness course or similar that incorporates different exercises that they have learnt how to do. They could video themselves in action and explain how to complete it etc* - *I would have liked bigger challenges* - *More structured exercises more often* - *Including parents in a competitive way may be fun* |
| **Technical difficulties** |
| - *Tracker kept running out of batteries and the app didnt always work, so became disjointed* - *The first tablet stopped working, we had to swap it. We also had lots of issues getting used to the layout. No back button, could not fix mistakes when made. He became disheartened when lots of his steps were not recorded and he could not access the prizes.* - *Program is easy - just the tech became an issue* - *Tablet stopped working. we lost thousands of steps in the meantime. No back button, no way to fix mistakes once entered. A parent really has to monitor the child doing it otherwise mistakes are made all the time.* - *Technology failing at different points during the study.* |
| **Dissatisfaction with activity tracker** |
| - *Maybe a friendly activity tracker that can be used during sport too* - *[child] couldn’t wear the tracker while training and playing rep soccer* - *Need to incorporate bike riding as tracker doesn't pick that up.* - *A different activity tracker like the Garmin - more reliable. More physical activities ([child] is almost 4 years chemo free). Maybe more communication with the facilitators.* - *We had lots of troubles with the tracker* - *Liked the whole program if only the tracker works efficiently* |
