# Supplementary material for: A Digital Educational Intervention With Wearable Activity Trackers to Support Health Behaviors Among Childhood Cancer Survivors: Pilot Feasibility and Acceptability Study
Source: JMIR Cancer. 2022 Aug 17;8(3):e38367. doi: 10.2196/38367 (PMC9434388; doi:10.2196/38367)
Supplement: Multimedia Appendix 3 [file cancer_v8i3e38367_app3.docx]

**Multimedia Appendix 3. Daily clusters**

| **Cluster groups (N=22)** | **MVPA** | | **SED** | |
| --- | --- | --- | --- | --- |
| **Cluster 1 (n=3)** | **≥ 3s** | **≥ 30s** | **≥ 60s** | **≥ 300s** |
| Total time (min) | 68.3 | 4.9 | 312.6 | 182.2 |
| Frequency of bouts | 637 | 7 | 77 | 11 |
| **Cluster 2 (n=3)** | | | | |
| Total time (min) | 47.2 | 2.4 | 192.7 | 138.8 |
| Frequency of bouts | 495 | 3 | 33 | 6 |
| **Cluster 3 (n=4)** | | | | |
| Total time (min) | 36.5 | 6.1 | 377.4 | 197.1 |
| Frequency of bouts | 336 | 6 | 109 | 9 |
| **Cluster 4 (n=10)** | | | | |
| Total time (min) | 26.3 | 1.1 | 319.3 | 129.6 |
| Frequency of bouts | 298 | 2 | 111 | 9 |
| **Cluster 5 (n=2)** | | | | |
| Total time (min) | 20.8 | 1.7 | 501.8 | 421.7 |
| Frequency of bouts | 245 | 2 | 57 | 13 |

**Abbreviations:** MVPA: moderate-to-vigorous physical activity; SED: sedentary activity
