# Supplementary material for: A Digital Educational Intervention With Wearable Activity Trackers to Support Health Behaviors Among Childhood Cancer Survivors: Pilot Feasibility and Acceptability Study
Source: JMIR Cancer. 2022 Aug 17;8(3):e38367. doi: 10.2196/38367 (PMC9434388; doi:10.2196/38367)
Supplement: Multimedia Appendix 4 [file cancer_v8i3e38367_app4.docx]

**Multimedia Appendix 4. Frequencies and proportions of reported health-related quality of life problems from the dichotomized EQ-5D-Y-5L.**

|  | **Pre-intervention (n=26)**  **n (%)** | **Post-intervention (n=8)**  **n (%)** | ***P* value** |
| --- | --- | --- | --- |
| **Mobility**  No problems  Any problems | 19 (73)  7 (27) | 8 (100)  0 (0) | 0.106 |
| **Self-care**  No problems  Any problems | 26 (100)  0 (0) | 8 (100)  0 (0) | 0.106 |
| **Activities of daily living**  No problems  Any problems | 19 (73)  7 (27) | 8 (100)  0 (0) | 0.106 |
| **Pain/discomfort**  No problems  Any problems | 12 (46)  14 (54) | 7 (88)  1 (12) | **0.040 *** |
| **Anxiety/depression**  No problems  Any problems | 17 (65)  9 (35) | 5 (63)  3 (28) | 0.886 |

* Significance at *P* < .05
