# Supplementary material for: A Digital Educational Intervention With Wearable Activity Trackers to Support Health Behaviors Among Childhood Cancer Survivors: Pilot Feasibility and Acceptability Study
Source: JMIR Cancer. 2022 Aug 17;8(3):e38367. doi: 10.2196/38367 (PMC9434388; doi:10.2196/38367)
Supplement: Multimedia Appendix 5 [file cancer_v8i3e38367_app5.docx]

**Multimedia Appendix 5. CONSORT-EHEALTH V1.6**

CONSORT-EHEALTH (V 1.6.1) - Submission/Publication

Form

The CONSORT-EHEALTH checklist is intended for authors of randomized trials evaluating web-based and Internet-based applications/interventions, including mobile interventions, electronic games (incl multiplayer games), social media, certain telehealth applications, and other interactive and/or networked electronic applications. Some of the items (e.g. all subitems under item 5 - description of the intervention) may also be applicable for other study designs.

The goal of the CONSORT EHEALTH checklist and guideline is to be a) a guide for reporting for authors of RCTs,

b) to form a basis for appraisal of an ehealth trial (in terms of validity)

CONSORT-EHEALTH items/subitems are MANDATORY reporting items for studies published in the Journal of Medical Internet Research and other journals / scientific societies endorsing the checklist.

Items numbered 1., 2., 3., 4a., 4b etc are original CONSORT or CONSORT-NPT (nonpharmacologic treatment) items.

Items with Roman numerals (i., ii, iii, iv etc.) are CONSORT-EHEALTH extensions/clarifications.

As the CONSORT-EHEALTH checklist is still considered in a formative stage, we would ask that you also RATE ON A SCALE OF 1-5 how important/useful you feel each item is FOR THE PURPOSE OF THE CHECKLIST and reporting guideline (optional).

Mandatory reporting items are marked with a red *.

In the textboxes, either copy & paste the relevant sections from your manuscript into this form - please include any quotes from your manuscript in QUOTATION MARKS, or answer directly by providing additional information not in the manuscript, or elaborating on why the item was not relevant for this study.

YOUR ANSWERS WILL BE PUBLISHED AS A SUPPLEMENTARY FILE TO YOUR PUBLICATION IN JMIR AND ARE CONSIDERED PART OF YOUR PUBLICATION (IF ACCEPTED).

Please fill in these questions diligently. Information will not be copyedited, so please use proper spelling and grammar, use correct capitalization, and avoid abbreviations.

DO NOT FORGET TO SAVE AS PDF _AND_ CLICK THE SUBMIT BUTTON SO YOUR ANSWERS ARE IN OUR DATABASE !!!

Citation Suggestion (if you append the pdf as Appendix we suggest to cite this paper in the caption):

Eysenbach G, CONSORT-EHEALTH Group

CONSORT-EHEALTH: Improving and Standardizing Evaluation Reports of Web-based and

Mobile Health Interventions

J Med Internet Res 2011;13(4):e126

URL: [http://www.jmir.org/2011/4/e126/](https://www.google.com/url?q=http://www.jmir.org/2011/4/e126/&sa=D&source=editors&ust=1648604086758234&usg=AOvVaw1oSXz9NFoEk9pT7bpJ2Yol)

doi: 10.2196/jmir.1923

PMID: 22209829

not shared

(

)

[t](https://accounts.google.com/AccountChooser?continue=https://docs.google.com/forms/d/e/1FAIpQLSfZBSUp1bwOc_OimqcS64RdfIAFvmrTSkZQL2-3O8O9hrL5Sw/viewform?hl%3Den_US%26formkey%3DdGlKd2Z2Q1lNSGQ0THl1azM5MS1aWWc6MA%26rm%3Dfull&service=wise)

[Switch accoun](https://accounts.google.com/AccountChooser?continue=https://docs.google.com/forms/d/e/1FAIpQLSfZBSUp1bwOc_OimqcS64RdfIAFvmrTSkZQL2-3O8O9hrL5Sw/viewform?hl%3Den_US%26formkey%3DdGlKd2Z2Q1lNSGQ0THl1azM5MS1aWWc6MA%26rm%3Dfull&service=wise)

Draft saved

* Required

Your name

*

First Last

Lauren Ha

Primary Affiliation (short), City, Country

*

University of Toronto, Toronto, Canada

UNSW Sydney, Sydney, Australia

Your e-mail address

*

abc

@g

mail.com

lauren.ha@unsw.edu.au

Title of your manuscript

*

Provide the (draft) title of your manuscript.

Feasibility and acceptability of “iBounce”: a digital educational intervention with wearable

activity trackers to support health behaviours among childhood cancer survivors

Name of your App/Software/Intervention

*

If there is a short and a long/alternate name, write the short name first and add the long name in

brackets.

iBounce

Evaluated Version (if any)

e.g. "V1",

"Release 2017-03-01", "Version 2.0.27913"

Your answer

Language(s)

*

What language is the intervention/app in? If multiple languages are available, separate by comma (e.g.

"English, French")

English

URL of your Intervention Website or App

e.g. a direct link to the mobile app on app in appstore (itunes, Google Play), or URL of the website. If the

intervention is a DVD or hardware, you can also link to an Amazon page.

Your answer

URL of an image/screenshot (optional)

Your answer

access is free and open

access only for special usergroups, not open

access is open to everyone, but requires payment/subscription/in-app purchases

app/intervention no longer accessible

Other:

Accessibility

*

Can an enduser access the intervention presently?

Primary Medical Indication/Disease/Condition

*

e.g. "Stress", "Diabetes", or define the target group in brackets after the condition, e.g. "Autism (Parents

of children with)", "Alzheimers (Informal Caregivers of)"

Cancer (childhood cancer survivors)

Primary Outcomes measured in trial

*

comma-separated list of primary outcomes reported in the trial

Feasibility, acceptability

Secondary/other outcomes

Are there any other outcomes the intervention is expected to affect?

Aerobic fitness levels, physical activity levels, health-related quality of life

Approximately Daily

Approximately Weekly

Approximately Monthly

Approximately Yearly

"as needed"

Other:

unknown / not evaluated

0-10

%

11-20

%

21-30

%

%

31-40

%

41-50

51-60

%

%

61-70

71

%-80%

81-90

%

91-100

%

Other:

Recommended "Dose"

*

What do the instructions for users say on how often the app should be used?

Approx. Percentage of Users (starters) still using the app as recommended after

3

months

*

yes: all primary outcomes were significantly better in intervention group vs control

partly: SOME primary outcomes were significantly better in intervention group vs

control

no statistically significant difference between control and intervention

potentially harmful: control was significantly better than intervention in one or more

outcomes

inconclusive: more research is needed

Other:

not submitted yet - in early draft status

not submitted yet - in late draft status, just before submission

submitted to a journal but not reviewed yet

submitted to a journal and after receiving initial reviewer comments

submitted to a journal and accepted, but not published yet

published

Other:

Overall, was the app/intervention effective?

*

Pilot intervention. No control group.

Article Preparation Status/Stage

*

At which stage in your article preparation are you currently (at the time you fill in this form)

not submitted yet / unclear where I will submit this

Journal of Medical Internet Research (JMIR)

JMIR mHealth and UHealth

JMIR Serious Games

JMIR Mental Health

JMIR Public Health

JMIR Formative Research

Other JMIR sister journal

Other:

Pilot/feasibility

Fully powered

no ms number (yet) / not (yet) submitted to / published in JMIR

Other:

TITLE AND ABSTRACT

Journal

*

If you already know where you will submit this paper (or if it is already submitted), please provide the

journal name (if it is not JMIR, provide the journal name under "other")

Is this a full powered effectiveness trial or a pilot/feasibility trial?

*

Manuscript tracking number

*

If this is a JMIR submission, please provide the manuscript tracking number under "other" (The ms

tracking number can be found in the submission acknowledgement email, or when you login as author in

JMIR. If the paper is already published in JMIR, then the ms tracking number is the four-digit number at

the end of the DOI, to be found at the bottom of each published article in JMIR)

1

a) TITLE: Identification as a randomized trial in the title

yes

Other:

subitem not at all important

1

2

3

4

5

essential

1

a) Does your paper address CONSORT item 1a?

*

I.e does the title contain the phrase "Randomized Controlled Trial"? (if not, explain the reason under

"other")

Pilot/feasibility study

1

a-i) Identify the mode of delivery in the title

Identify the mode of delivery. Preferably use “web-based” and/or “mobile” and/or “electronic game” in the

title. Avoid ambiguous terms like “online”, “virtual”, “interactive”. Use “Internet-based” only if Intervention

includes non-web-based Internet components (e.g. email), use “computer-based” or “electronic” only if

offline products are used. Use “virtual” only in the context of “virtual reality” (3-D worlds). Use “online”

only in the context of “online support groups”. Complement or substitute product names with broader

terms for the class of products (such as “mobile” or “smart phone” instead of “iphone”), especially if the

application runs on different platforms.

Clear selection

Does your paper address subitem 1a-i?

*

Copy and paste relevant sections from manuscript title (include quotes in quotation marks "like this"

to

indicate direct quotes from your manuscript), or elaborate on this item by providing additional

information not in the ms, or briefly explain why the item is not applicable/relevant for your study

"a digital educational intervention"

subitem not at all important

1

2

3

4

5

essential

subitem not at all important

1

2

3

4

5

essential

1

a-ii) Non-web-based components or important co-interventions in title

Mention non-web-based components or important co-interventions in title, if any (e.g., “with telephone

support”).

Clear selection

Does your paper address subitem 1a-ii?

Copy and paste relevant sections from manuscript title (include quotes in quotation marks "like this"

to

indicate direct quotes from your manuscript), or elaborate on this item by providing additional

information not in the ms, or briefly explain why the item is not applicable/relevant for your study

"with wearable activity trackers"

1

a-iii) Primary condition or target group in the title

Mention primary condition or target group in the title, if any (e.g., “for children with Type I Diabetes”)

Example: A Web-based and Mobile Intervention with Telephone Support for Children with Type I Diabetes:

Randomized Controlled Trial

Clear selection

Does your paper address subitem 1a-iii?

*

Copy and paste relevant sections from manuscript title (include quotes in quotation marks "like this"

to

indicate direct quotes from your manuscript), or elaborate on this item by providing additional

information not in the ms, or briefly explain why the item is not applicable/relevant for your study

"childhood cancer survivors"

b) ABSTRACT: Structured summary of trial design, methods, results, and

1

conclusions

NPT extension: Description of experimental treatment, comparator, care providers, centers, and blinding

status.

subitem not at all important

1

2

3

4

5

essential

subitem not at all important

1

2

3

4

5

essential

1

b-i) Key features/functionalities/components of the intervention and

comparator in the METHODS section of the ABSTRACT

Mention key features/functionalities/components of the intervention and comparator in the abstract. If

possible, also mention theories and principles used for designing the site. Keep in mind the needs of

systematic reviewers and indexers by including important synonyms. (Note: Only report in the abstract

what the main paper is reporting. If this information is missing from the main body of text, consider

adding it)

Clear selection

Does your paper address subitem 1b-i?

*

Copy and paste relevant sections from the manuscript abstract (include quotes in quotation marks "like

this"

to indicate direct quotes from your manuscript), or elaborate on this item by providing additional

information not in the ms, or briefly explain why the item is not applicable/relevant for your study

"12-week self-guided online program (adapted from iEngage©) involved 10 educational

modules, goal-setting, and home-based physical activities monitored using an activity

tracker (Misfit Ray©)."

1

b-ii) Level of human involvement in the METHODS section of the ABSTRACT

Clarify the level of human involvement in the abstract, e.g., use phrases like “fully automated” vs.

“therapist/nurse/care provider/physician-assisted” (mention number and expertise of providers involved,

if any). (Note: Only report in the abstract what the main paper is reporting. If this information is missing

from the main body of text, consider adding it)

Clear selection

Does your paper address subitem 1b-ii?

Copy and paste relevant sections from the manuscript abstract (include quotes in quotation marks "like this" to indicate direct quotes from your manuscript), or elaborate on this item by providing additional information not in the ms, or briefly explain why the item is not applicable/relevant for your study

"12-week self-guided online program"

# 1b-iii) Open vs. closed, web-based (self-assessment) vs. face-to-face assessments in the METHODS section of the ABSTRACT

Mention how participants were recruited (online vs. offline), e.g., from an open access website or from a clinic or a closed online user group (closed usergroup trial), and clarify if this was a purely web-based trial, or there were face-to-face components (as part of the intervention or for assessment). Clearly say if outcomes were self-assessed through questionnaires (as common in web-based trials). Note: In traditional offline trials, an open trial (open-label trial) is a type of clinical trial in which both the researchers and participants know which treatment is being administered. To avoid confusion, use “blinded” or “unblinded” to indicated the level of blinding instead of “open”, as “open” in web-based trials usually refers to “open access” (i.e. participants can self-enrol). (Note: Only report in the abstract what the main paper is reporting. If this information is missing from the main body of text, consider adding it)

1 2 3 4 5

subitem not at all important essential

Clear selection

Does your paper address subitem 1b-iii?

Copy and paste relevant sections from the manuscript abstract (include quotes in quotation marks "like this" to indicate direct quotes from your manuscript), or elaborate on this item by providing additional information not in the ms, or briefly explain why the item is not applicable/relevant for your study

"We recruited 8–13-year-old survivors who were ≥12 months post-cancer treatment completion. The 12-week self-guided online program (adapted from iEngage©) involved 10 educational modules, goal-setting, and home-based physical activities monitored using an activity tracker (Misfit Ray©). We assessed objective physical activity levels (GeneActiv accelerometer) and behaviours using cluster analysis, aerobic fitness (6-minute walk test), and HRQOL (EQ-5D-Y-5L) at baseline and post-intervention (week 12). Parents were trained to reassess aerobic fitness at home at follow-up (week 24). "

https://docs.google.com/forms/d/e/1FAIpQLSfZBSUp1bwOc_OimqcS64RdfIAFvmrTSkZQL2-3O8O9hrL5Sw/viewform?hl=en_US&formkey=dGl…

subitem not at all important

1

2

3

4

5

essential

subitem not at all important

1

2

3

4

5

essential

1

b-iv) RESULTS section in abstract must contain use data

Report number of participants enrolled/assessed in each group, the use/uptake of the intervention (e.g.,

attrition/adherence metrics, use over time, number of logins etc.), in addition to primary/secondary

outcomes. (Note: Only report in the abstract what the main paper is reporting. If this information is

missing from the main body of text, consider adding it)

Clear selection

Does your paper address subitem 1b-iv?

Copy and paste relevant sections from the manuscript abstract (include quotes in quotation marks "like

this"

to indicate direct quotes from your manuscript), or elaborate on this item by providing additional

information not in the ms, or briefly explain why the item is not applicable/relevant for your study

"Thirty participants opted in, 27 completed baseline assessments and 23 commenced

iBounce. "

1

b-v) CONCLUSIONS/DISCUSSION in abstract for negative trials

Conclusions/Discussions in abstract for negative trials: Discuss the primary outcome - if the trial is

negative (primary outcome not changed), and the intervention was not used, discuss whether negative

results are attributable to lack of uptake and discuss reasons. (Note: Only report in the abstract what the

main paper is reporting. If this information is missing from the main body of text, consider adding it)

Clear selection

INTRODUCTION

2

a) In INTRODUCTION: Scientific background and explanation of rationale

subitem not at all important

1

2

3

4

5

essential

Does your paper address subitem 1b-v?

Copy and paste relevant sections from the manuscript abstract (include quotes in quotation marks "like

this"

to indicate direct quotes from your manuscript), or elaborate on this item by providing additional

information not in the ms, or briefly explain why the item is not applicable/relevant for your study

"We have demonstrated iBounce to be feasible to deliver and acceptable among survivors,

despite some technical difficulties. The distance-delivered format provides an opportunity

to engage survivors in physical activity at home and may address barriers to care,

particularly for regional or remote families. We will use these pilot findings to enhance and

evaluate an updated version of iBounce."

2

a-i) Problem and the type of system/solution

Describe the problem and the type of system/solution that is object of the study: intended as stand-alone

intervention vs. incorporated in broader health care program? Intended for a particular patient

population? Goals of the intervention, e.g., being more cost-effective to other interventions, replace or

complement other solutions? (Note: Details about the intervention are provided in “Methods” under 5)

Clear selection

Does your paper address subitem 2a-i?

*

Copy and paste relevant sections from the manuscript (include quotes in quotation marks "like this"

to

indicate direct quotes from your manuscript), or elaborate on this item by providing additional

information not in the ms, or briefly explain why the item is not applicable/relevant for your study

"the delivery of health behaviour interventions using distance-delivered technologies is a

growing field to address physical inactivity and low fitness levels, and improve access to

services among this population", "This study aimed to pilot ‘iBounce’, a distance-delivered

health education intervention to foster health behaviours (i.e., physical activity to improve

fitness levels) among survivors of childhood cancer."

# 2a-ii) Scientific background, rationale: What is known about the (type of) system

Scientific background, rationale: What is known about the (type of) system that is the object of the study (be sure to discuss the use of similar systems for other conditions/diagnoses, if appropiate), motivation for the study, i.e. what are the reasons for and what is the context for this specific study, from which stakeholder viewpoint is the study performed, potential impact of findings [2]. Briefly justify the choice of the comparator.

1 2 3 4 5

subitem not at all important essential

# Does your paper address subitem 2a-ii? *

Copy and paste relevant sections from the manuscript (include quotes in quotation marks "like this" to indicate direct quotes from your manuscript), or elaborate on this item by providing additional information not in the ms, or briefly explain why the item is not applicable/relevant for your study

"Health behaviour interventions, including physical activity promotion and engagement is crucial for preventing or minimising the impact of these late effects5,6. Additionally, physical activity may have a positive influence on survivors’ health-related quality of life (HRQOL)7. Yet, many survivors do not engage in sufficient levels of physical activity8, have poor perceptions of their activity levels9, and have below average fitness levels10", "When surveyed, 60% of survivors expressed a need for age-appropriate exercise information, and 79% reported a desire for exercise guidance11. A major burden for many survivors and families, especially for the 45% living in rural or regional areas in Australia, is the tyranny of distance, compounded by the financial burden of travel and accommodation12. As a result, families from rural and regional areas have less access to supportive care, experience greater cancer-related financial hardship in survivorship compared to metropolitan families, and are therefore at highest risk of poor health outcomes13,14. Consequently, the delivery of health behaviour interventions using distance-delivered technologies is a growing field to address physical inactivity and low fitness levels, and improve access to services among this population15-17. "

# 2b) In INTRODUCTION: Specific objectives or hypotheses

METHODS

a) Description of trial design (such as parallel, factorial) including allocation

3

ratio

3

b) Important changes to methods after trial commencement (such as

eligibility criteria), with reasons

Does your paper address CONSORT subitem 2b?

*

Copy and paste relevant sections from the manuscript (include quotes in quotation marks "like this"

to

indicate direct quotes from your manuscript), or elaborate on this item by providing additional

information not in the ms, or briefly explain why the item is not applicable/relevant for your study

"Our primary aim of this study was to evaluate the feasibility and acceptability to survivors

of using iBounce at home. Our secondary aims were to assess the impact of the iBounce

intervention on survivors’ physical activity levels and behaviours, aerobic fitness, and

HRQOL."

Does your paper address CONSORT subitem 3a?

*

Copy and paste relevant sections from the manuscript (include quotes in quotation marks "like this"

to

indicate direct quotes from your manuscript), or elaborate on this item by providing additional

information not in the ms, or briefly explain why the item is not applicable/relevant for your study

N/A - pilot feasibility study.

Does your paper address CONSORT subitem 3b?

*

Copy and paste relevant sections from the manuscript (include quotes in quotation marks "like this"

to

indicate direct quotes from your manuscript), or elaborate on this item by providing additional

information not in the ms, or briefly explain why the item is not applicable/relevant for your study

N/A - nil changes were made to methods after trial commencement.

subitem not at all important

1

2

3

4

5

essential

a) Eligibility criteria for participants

4

3

b-i) Bug fixes, Downtimes, Content Changes

Bug fixes, Downtimes, Content Changes: ehealth systems are often dynamic systems. A description of

changes to methods therefore also includes important changes made on the intervention or comparator

during the trial (e.g., major bug fixes or changes in the functionality or content) (5-iii) and other

“unexpected events” that may have influenced study design such as staff changes, system

failures/downtimes, etc. [2].

Clear selection

Does your paper address subitem 3b-i?

Copy and paste relevant sections from the manuscript (include quotes in quotation marks "like this"

to

indicate direct quotes from your manuscript), or elaborate on this item by providing additional

information not in the ms, or briefly explain why the item is not applicable/relevant for your study

N/A - nil changes to methods

Does your paper address CONSORT subitem 4a?

*

Copy and paste relevant sections from the manuscript (include quotes in quotation marks "like this"

to

indicate direct quotes from your manuscript), or elaborate on this item by providing additional

information not in the ms, or briefly explain why the item is not applicable/relevant for your study

"Our eligibility criteria included participants who were (i) aged 8-13 years old, (ii) diagnosed

with childhood cancer, (iii)

≥

12

months post cancer therapy completion or undergoing

maintenance chemotherapy, (iv) able to communicate in English, and (v) had internet access

at home. Participants were excluded if they (i) had cancer relapse after recruitment, (ii) had

a medical condition that would prohibit exercise, (iii) were participating in another research

study that would affect the study primary and secondary outcomes, or (iv) had previously

completed a research study <4 weeks prior. "

subitem not at all important

1

2

3

4

5

essential

subitem not at all important

1

2

3

4

5

essential

4

a-i) Computer / Internet literacy

Computer / Internet literacy is often an implicit “de facto” eligibility criterion - this should be explicitly

clarified.

Clear selection

Does your paper address subitem 4a-i?

Copy and paste relevant sections from the manuscript (include quotes in quotation marks "like this"

to

indicate direct quotes from your manuscript), or elaborate on this item by providing additional

information not in the ms, or briefly explain why the item is not applicable/relevant for your study

N/A Not relevant to the study

4

a-ii) Open vs. closed, web-based vs. face-to-face assessments

:

Open vs. closed, web-based vs. face-to-face assessments: Mention how participants were recruited

(

online vs. offline), e.g., from an open access website or from a clinic, and clarify if this was a purely web

-

based trial, or there were face-to-face components (as part of the intervention or for assessment), i.e., to

what degree got the study team to know the participant. In online-only trials, clarify if participants were

quasi-anonymous and whether having multiple identities was possible or whether technical or logistical

measures (e.g., cookies, email confirmation, phone calls) were used to detect/prevent these.

Clear selection

subitem not at all important

1

2

3

4

5

essential

b) Settings and locations where the data were collected

4

Does your paper address subitem 4a-ii?

*

Copy and paste relevant sections from the manuscript (include quotes in quotation marks "like this"

to

indicate direct quotes from your manuscript), or elaborate on this item by providing additional

information not in the ms, or briefly explain why the item is not applicable/relevant for your study

"Nursing staff identified eligible participants through hospital clinic lists with approval from

treating oncologists. The study coordinator (LH) telephoned eligible parents or carers of

survivors (henceforth called “parents”) to discuss the study with the study invitation

package sent via email or post. An initial consultation was organised with consenting

parents and survivors, and study equipment were provided for participants. After written

informed consent was provided by parents, survivors were enrolled in the study."

4

a-iii) Information giving during recruitment

Information given during recruitment. Specify how participants were briefed for recruitment and in the

informed consent procedures (e.g., publish the informed consent documentation as appendix, see also

item X26), as this information may have an effect on user self-selection, user expectation and may also

bias results.

Clear selection

Does your paper address subitem 4a-iii?

Copy and paste relevant sections from the manuscript (include quotes in quotation marks "like this"

to

indicate direct quotes from your manuscript), or elaborate on this item by providing additional

information not in the ms, or briefly explain why the item is not applicable/relevant for your study

"An initial consultation was organised with consenting parents and survivors, and study

equipment were provided for participants. After written informed consent was provided by

parents, survivors were enrolled in the study."

subitem not at all important

1

2

3

4

5

essential

subitem not at all important

1

2

3

4

5

essential

Does your paper address CONSORT subitem 4b?

*

Copy and paste relevant sections from the manuscript (include quotes in quotation marks "like this"

to

indicate direct quotes from your manuscript), or elaborate on this item by providing additional

information not in the ms, or briefly explain why the item is not applicable/relevant for your study

"We recruited participants from Sydney Children’s Hospital, Australia, between May 2019

and May 2021."

4

b-i) Report if outcomes were (self-)assessed through online questionnaires

Clearly report if outcomes were (self-)assessed through online questionnaires (as common in web-based

trials) or otherwise.

Clear selection

Does your paper address subitem 4b-i?

*

Copy and paste relevant sections from the manuscript (include quotes in quotation marks "like this"

to

indicate direct quotes from your manuscript), or elaborate on this item by providing additional

information not in the ms, or briefly explain why the item is not applicable/relevant for your study

"We used the EQ-5D-Y-5L tool to assess self-reported HRQOL in survivors at baseline and 12

weeks post-intervention"

4

b-ii) Report how institutional affiliations are displayed

Report how institutional affiliations are displayed to potential participants [on ehealth media], as

affiliations with prestigious hospitals or universities may affect volunteer rates, use, and reactions with

regards to an intervention.(Not a required item – describe only if this may bias results)

5)

The interventions for each group with sufficient details to allow replication,

including how and when they were actually administered

subitem not at all important

1

2

3

4

5

essential

Does your paper address subitem 4b-ii?

Copy and paste relevant sections from the manuscript (include quotes in quotation marks "like this"

to

indicate direct quotes from your manuscript), or elaborate on this item by providing additional

information not in the ms, or briefly explain why the item is not applicable/relevant for your study

Your answer

5-

i) Mention names, credential, affiliations of the developers, sponsors, and

owners

Mention names, credential, affiliations of the developers, sponsors, and owners [6] (if authors/evaluators

are owners or developer of the software, this needs to be declared in a “Conflict of interest” section or

mentioned elsewhere in the manuscript).

Clear selection

Does your paper address subitem 5-i?

Copy and paste relevant sections from the manuscript (include quotes in quotation marks "like this"

to

indicate direct quotes from your manuscript), or elaborate on this item by providing additional

information not in the ms, or briefly explain why the item is not applicable/relevant for your study

"The iBounce program uses iEngage©, built by BePatient, a company that provides flexible

and modular patient-centric e-health platforms for health care and research"

subitem not at all important

1

2

3

4

5

essential

subitem not at all important

1

2

3

4

5

essential

5-

ii) Describe the history/development process

Describe the history/development process of the application and previous formative evaluations (e.g.,

focus groups, usability testing), as these will have an impact on adoption/use rates and help with

interpreting results.

Clear selection

Does your paper address subitem 5-ii?

Copy and paste relevant sections from the manuscript (include quotes in quotation marks "like this"

to

indicate direct quotes from your manuscript), or elaborate on this item by providing additional

information not in the ms, or briefly explain why the item is not applicable/relevant for your study

"iEngage© was built for 10-12 year old school children without a chronic disease, and was

piloted in a rural school in New Caledonia18 and trialed in two primary schools in Sydney,

Australia in 2017 and 2018. Caillaud et al. and Diaz et al. detail the iEngage© program. "

5-

iii) Revisions and updating

Revisions and updating. Clearly mention the date and/or version number of the application/intervention

(

and comparator, if applicable) evaluated, or describe whether the intervention underwent major changes

during the evaluation process, or whether the development and/or content was “frozen” during the trial.

Describe dynamic components such as news feeds or changing content which may have an impact on

the replicability of the intervention (for unexpected events see item 3b).

Does your paper address subitem 5-iii?

Copy and paste relevant sections from the manuscript (include quotes in quotation marks "like this"

to

indicate direct quotes from your manuscript), or elaborate on this item by providing additional

information not in the ms, or briefly explain why the item is not applicable/relevant for your study

Your answer

subitem not at all important

1

2

3

4

5

essential

subitem not at all important

1

2

3

4

5

essential

5-

iv) Quality assurance methods

Provide information on quality assurance methods to ensure accuracy and quality of information

provided [1], if applicable.

Does your paper address subitem 5-iv?

Copy and paste relevant sections from the manuscript (include quotes in quotation marks "like this"

to

indicate direct quotes from your manuscript), or elaborate on this item by providing additional

information not in the ms, or briefly explain why the item is not applicable/relevant for your study

Your answer

5-

v) Ensure replicability by publishing the source code, and/or providing

screenshots/screen-capture video, and/or providing flowcharts of the algorithms

used

Ensure replicability by publishing the source code, and/or providing screenshots/screen-capture video,

and/or providing flowcharts of the algorithms used. Replicability (i.e., other researchers should in

principle be able to replicate the study) is a hallmark of scientific reporting.

Clear selection

Does your paper address subitem 5-v?

Copy and paste relevant sections from the manuscript (include quotes in quotation marks "like this"

to

indicate direct quotes from your manuscript), or elaborate on this item by providing additional

information not in the ms, or briefly explain why the item is not applicable/relevant for your study

"We performed our cluster analysis using R (R Studio), published in Open Science

Framework (https://osf.io/ng3tk/). "

subitem not at all important

1

2

3

4

5

essential

subitem not at all important

1

2

3

4

5

essential

5-

vi) Digital preservation

Digital preservation: Provide the URL of the application, but as the intervention is likely to change or

disappear over the course of the years; also make sure the intervention is archived (Internet Archive,

[webcitation.or](https://www.google.com/url?q=http://webcitation.org&sa=D&source=editors&ust=1648604086805902&usg=AOvVaw2nE44SHsAX8yxmaX59R76L)

[g](https://www.google.com/url?q=http://webcitation.org&sa=D&source=editors&ust=1648604086805902&usg=AOvVaw2nE44SHsAX8yxmaX59R76L)

and/or publishing the source code or screenshots/videos alongside the article). As

[,](https://www.google.com/url?q=http://webcitation.org&sa=D&source=editors&ust=1648604086805902&usg=AOvVaw2nE44SHsAX8yxmaX59R76L)

pages behind login screens cannot be archived, consider creating demo pages which are accessible

without login.

Does your paper address subitem 5-vi?

Copy and paste relevant sections from the manuscript (include quotes in quotation marks "like this"

to

indicate direct quotes from your manuscript), or elaborate on this item by providing additional

information not in the ms, or briefly explain why the item is not applicable/relevant for your study

Your answer

5-

vii) Access

Access: Describe how participants accessed the application, in what setting/context, if they had to pay

(

or were paid) or not, whether they had to be a member of specific group. If known, describe how

participants obtained “access to the platform and Internet” [1]. To ensure access for

editors/reviewers/readers, consider to provide a “backdoor” login account or demo mode for

reviewers/readers to explore the application (also important for archiving purposes, see vi).

Clear selection

Does your paper address subitem 5-vii?

*

Copy and paste relevant sections from the manuscript (include quotes in quotation marks "like this"

to

indicate direct quotes from your manuscript), or elaborate on this item by providing additional

information not in the ms, or briefly explain why the item is not applicable/relevant for your study

"After written informed consent was provided by parents, survivors were enrolled in the

study and provided with account login details to the program. "

# 5-viii) Mode of delivery, features/functionalities/components of the intervention and comparator, and the theoretical framework

Describe mode of delivery, features/functionalities/components of the intervention and comparator, and the theoretical framework [6] used to design them (instructional strategy [1], behaviour change techniques, persuasive features, etc., see e.g., [7, 8] for terminology). This includes an in-depth description of the content (including where it is coming from and who developed it) [1],” whether [and how] it is tailored to individual circumstances and allows users to track their progress and receive feedback” [6]. This also includes a description of communication delivery channels and – if computermediated communication is a component – whether communication was synchronous or asynchronous [6]. It also includes information on presentation strategies [1], including page design principles, average amount of text on pages, presence of hyperlinks to other resources, etc. [1].

1 2 3 4 5

subitem not at all important essential

Clear selection

# Does your paper address subitem 5-viii? *

Copy and paste relevant sections from the manuscript (include quotes in quotation marks "like this" to indicate direct quotes from your manuscript), or elaborate on this item by providing additional information not in the ms, or briefly explain why the item is not applicable/relevant for your study

". The iEngage© digital app includes animated animal characters who guide the child through 10 self-paced educational modules which focus on different health topics including physical activity, muscular strength, sedentary behaviours, and fitness (see Multimedia Appendix 1 for a summary of the module topics). There is also a focus on health literacy throughout the program teaching participants to define and classify physical activity intensities (light, moderate, and vigorous), self-ratings of perceived effort during exercise, recommended guidelines relating to physical activity, sedentary behaviours, fitness, wellbeing (physical, mental, and social), screen-based behaviours, and sugar intake. iEngage© was built for 10-12 year old school children without a chronic disease, and was piloted in a rural school in New Caledonia18 and trialed in two primary schools in Sydney, Australia in 2017 and 201819,21. Caillaud et al.19 and Diaz et al.21 detail the iEngage© program. " "For this study, we adapted iEngage© by (i) modifying the activities so that they were suitable for completion in a home-setting, (ii) modifying the module contents to encourage participation with family and friends, (iii) expanding readability to ensure younger children could understand the content, (iv) including a messaging platform for participants to contact the study coordinator for technical assistance or general inquiries, and (v) educating parents on how to assess and administer an aerobic fitness test for their child. We aimed to target children aged 8-13 years old as this is a critical life period for a child, when they are learning habits and becoming more independent22. "

subitem not at all important

1

2

3

4

5

essential

subitem not at all important

1

2

3

4

5

essential

5-

ix) Describe use parameters

Describe use parameters (e.g., intended “doses” and optimal timing for use). Clarify what instructions or

recommendations were given to the user, e.g., regarding timing, frequency, heaviness of use, if any, or

was the intervention used ad libitum.

Clear selection

Does your paper address subitem 5-ix?

Copy and paste relevant sections from the manuscript (include quotes in quotation marks "like this"

to

indicate direct quotes from your manuscript), or elaborate on this item by providing additional

information not in the ms, or briefly explain why the item is not applicable/relevant for your study

"We recommended for participants to complete the modules once or twice per week, and to

synchronise their activity tracker to the program daily."

5-

x) Clarify the level of human involvement

Clarify the level of human involvement (care providers or health professionals, also technical assistance)

in the e-intervention or as co-intervention (detail number and expertise of professionals involved, if any,

as well as “type of assistance offered, the timing and frequency of the support, how it is initiated, and the

medium by which the assistance is delivered”. It may be necessary to distinguish between the level of

human involvement required for the trial, and the level of human involvement required for a routine

application outside of a RCT setting (discuss under item 21 – generalizability).

Does your paper address subitem 5-x?

Copy and paste relevant sections from the manuscript (include quotes in quotation marks "like this"

to

indicate direct quotes from your manuscript), or elaborate on this item by providing additional

information not in the ms, or briefly explain why the item is not applicable/relevant for your study

"messaging platform for participants to contact the study coordinator for technical

assistance or general inquiries"

subitem not at all important

1

2

3

4

5

essential

subitem not at all important

1

2

3

4

5

essential

5-

xi) Report any prompts/reminders used

Report any prompts/reminders used: Clarify if there were prompts (letters, emails, phone calls, SMS) to

use the application, what triggered them, frequency etc. It may be necessary to distinguish between the

level of prompts/reminders required for the trial, and the level of prompts/reminders for a routine

application outside of a RCT setting (discuss under item 21 – generalizability).

Clear selection

Does your paper address subitem 5-xi?

*

Copy and paste relevant sections from the manuscript (include quotes in quotation marks "like this"

to

indicate direct quotes from your manuscript), or elaborate on this item by providing additional

information not in the ms, or briefly explain why the item is not applicable/relevant for your study

Prompts and reminders not appplicable to the study.

5-

xii) Describe any co-interventions (incl. training/support

)

Describe any co-interventions (incl. training/support): Clearly state any interventions that are provided in

addition to the targeted eHealth intervention, as ehealth intervention may not be designed as stand-alone

intervention. This includes training sessions and support [1]. It may be necessary to distinguish between

the level of training required for the trial, and the level of training for a routine application outside of a

RCT setting (discuss under item 21 – generalizability.

Does your paper address subitem 5-xii?

*

Copy and paste relevant sections from the manuscript (include quotes in quotation marks "like this"

to

indicate direct quotes from your manuscript), or elaborate on this item by providing additional

information not in the ms, or briefly explain why the item is not applicable/relevant for your study

Co-interventions not applicable to the study.

6

a) Completely defined pre-specified primary and secondary outcome

measures, including how and when they were assessed

subitem not at all important

1

2

3

4

5

essential

Does your paper address CONSORT subitem 6a?

*

Copy and paste relevant sections from the manuscript (include quotes in quotation marks "like this"

to

indicate direct quotes from your manuscript), or elaborate on this item by providing additional

information not in the ms, or briefly explain why the item is not applicable/relevant for your study

"Survivors and parents completed questionnaires at baseline and post-intervention (week

12)

. We assessed survivors’ objective physical activity levels and aerobic fitness

assessments at baseline and after completing the iBounce intervention. Aerobic fitness was

assessed by parents at home at follow-up (week 24)."

6

a-i) Online questionnaires: describe if they were validated for online use and

apply CHERRIES items to describe how the questionnaires were

designed/deployed

If outcomes were obtained through online questionnaires, describe if they were validated for online use

and apply CHERRIES items to describe how the questionnaires were designed/deployed [9].

Does your paper address subitem 6a-i?

Copy and paste relevant sections from manuscript text

"The EQ-5D-Y-5L has been validated in children aged 8-16 years with paediatric conditions,

including childhood cancer with a test-retest reliability of 0.84"

subitem not at all important

1

2

3

4

5

essential

subitem not at all important

1

2

3

4

5

essential

b) Any changes to trial outcomes after the trial commenced, with reasons

6

6

a-ii) Describe whether and how “use” (including intensity of use/dosage) was

defined/measured/monitored

Describe whether and how “use” (including intensity of use/dosage) was defined/measured/monitored

logins, logfile analysis, etc.). Use/adoption metrics are important process outcomes that should be

(

reported in any ehealth trial.

Does your paper address subitem 6a-ii?

Copy and paste relevant sections from manuscript text

Your answer

6

a-iii) Describe whether, how, and when qualitative feedback from participants

was obtained

Describe whether, how, and when qualitative feedback from participants was obtained (e.g., through

emails, feedback forms, interviews, focus groups).

Does your paper address subitem 6a-iii?

Copy and paste relevant sections from manuscript text

"At 12-weeks post-intervention, survivors completed an acceptability questionnaire which

included items adapted from the Youth Satisfaction Questionnaire (YSQ)25 in addition to

four purposely designed items to measure survivors’ enjoyment and satisfaction with the

iBounce program using a 0-100 scale (0=not at all, 100=enjoyed lots), and open-text fields to

assess reasons for their satisfaction rating. "

7

a) How sample size was determined

NPT: When applicable, details of whether and how the clustering by care provides or centers was

addressed

subitem not at all important

1

2

3

4

5

essential

7

b) When applicable, explanation of any interim analyses and stopping

guidelines

Does your paper address CONSORT subitem 6b?

*

Copy and paste relevant sections from the manuscript (include quotes in quotation marks "like this"

to

indicate direct quotes from your manuscript), or elaborate on this item by providing additional

information not in the ms, or briefly explain why the item is not applicable/relevant for your study

not applicable to the study.

7

a-i) Describe whether and how expected attrition was taken into account when

calculating the sample size

Describe whether and how expected attrition was taken into account when calculating the sample size.

Clear selection

Does your paper address subitem 7a-i?

Copy and paste relevant sections from manuscript title (include quotes in quotation marks "like this"

to

indicate direct quotes from your manuscript), or elaborate on this item by providing additional

information not in the ms, or briefly explain why the item is not applicable/relevant for your study

Your answer

8

a) Method used to generate the random allocation sequence

NPT: When applicable, how care providers were allocated to each trial group

b) Type of randomisation; details of any restriction (such as blocking and

8

block size)

9)

Mechanism used to implement the random allocation sequence (such as

sequentially numbered containers), describing any steps taken to conceal the

sequence until interventions were assigned

Does your paper address CONSORT subitem 7b?

*

Copy and paste relevant sections from the manuscript (include quotes in quotation marks "like this"

to

indicate direct quotes from your manuscript), or elaborate on this item by providing additional

information not in the ms, or briefly explain why the item is not applicable/relevant for your study

Not applicable to the study.

Does your paper address CONSORT subitem 8a?

*

Copy and paste relevant sections from the manuscript (include quotes in quotation marks "like this"

to

indicate direct quotes from your manuscript), or elaborate on this item by providing additional

information not in the ms, or briefly explain why the item is not applicable/relevant for your study

Not applicable to the study.

Does your paper address CONSORT subitem 8b?

*

Copy and paste relevant sections from the manuscript (include quotes in quotation marks "like this"

to

indicate direct quotes from your manuscript), or elaborate on this item by providing additional

information not in the ms, or briefly explain why the item is not applicable/relevant for your study

Not applicable to the study.

10)

Who generated the random allocation sequence, who enrolled

participants, and who assigned participants to interventions

a) If done, who was blinded after assignment to interventions (for example,

11

participants, care providers, those assessing outcomes) and how

NPT: Whether or not administering co-interventions were blinded to group assignment

subitem not at all important

1

2

3

4

5

essential

Does your paper address CONSORT subitem 9?

*

Copy and paste relevant sections from the manuscript (include quotes in quotation marks "like this"

to

indicate direct quotes from your manuscript), or elaborate on this item by providing additional

information not in the ms, or briefly explain why the item is not applicable/relevant for your study

Not applicable to the study.

Does your paper address CONSORT subitem 10?

*

Copy and paste relevant sections from the manuscript (include quotes in quotation marks "like this"

to

indicate direct quotes from your manuscript), or elaborate on this item by providing additional

information not in the ms, or briefly explain why the item is not applicable/relevant for your study

"Nursing staff identified eligible participants through hospital clinic lists with approval from

treating oncologists. The study coordinator (LH) telephoned eligible parents or carers of

survivors (henceforth called “parents”) to discuss the study with the study invitation

package sent via email or post. "

11

a-i) Specify who was blinded, and who wasn’t

Specify who was blinded, and who wasn’t. Usually, in web-based trials it is not possible to blind the

participants [1, 3] (this should be clearly acknowledged), but it may be possible to blind outcome

assessors, those doing data analysis or those administering co-interventions (if any).

subitem not at all important

1

2

3

4

5

essential

11

b) If relevant, description of the similarity of interventions

(

this item is usually not relevant for ehealth trials as it refers to similarity of a placebo or sham

intervention to a active medication/intervention)

Does your paper address subitem 11a-i?

*

Copy and paste relevant sections from the manuscript (include quotes in quotation marks "like this"

to

indicate direct quotes from your manuscript), or elaborate on this item by providing additional

information not in the ms, or briefly explain why the item is not applicable/relevant for your study

Not applicable to the study.

11

a-ii) Discuss e.g., whether participants knew which intervention was the

“intervention of interest” and which one was the “comparator”

Informed consent procedures (4a-ii) can create biases and certain expectations - discuss e.g., whether

participants knew which intervention was the “intervention of interest” and which one was the

“comparator”.

Does your paper address subitem 11a-ii?

Copy and paste relevant sections from the manuscript (include quotes in quotation marks "like this"

to

indicate direct quotes from your manuscript), or elaborate on this item by providing additional

information not in the ms, or briefly explain why the item is not applicable/relevant for your study

Your answer

Does your paper address CONSORT subitem 11b?

*

Copy and paste relevant sections from the manuscript (include quotes in quotation marks "like this"

to

indicate direct quotes from your manuscript), or elaborate on this item by providing additional

information not in the ms, or briefly explain why the item is not applicable/relevant for your study

Not applicable to the study.

12

a) Statistical methods used to compare groups for primary and secondary

outcomes

NPT: When applicable, details of whether and how the clustering by care providers or centers was

addressed

subitem not at all important

1

2

3

4

5

essential

b) Methods for additional analyses, such as subgroup analyses and adjusted

12

analyses

Does your paper address CONSORT subitem 12a?

*

Copy and paste relevant sections from the manuscript (include quotes in quotation marks "like this"

to

indicate direct quotes from your manuscript), or elaborate on this item by providing additional

information not in the ms, or briefly explain why the item is not applicable/relevant for your study

Not applicable to the study.

a-i) Imputation techniques to deal with attrition / missing values

12

Imputation techniques to deal with attrition / missing values: Not all participants will use the

intervention/comparator as intended and attrition is typically high in ehealth trials. Specify how

participants who did not use the application or dropped out from the trial were treated in the statistical

analysis (a complete case analysis is strongly discouraged, and simple imputation techniques such as

LOCF may also be problematic [4]).

Does your paper address subitem 12a-i?

*

Copy and paste relevant sections from the manuscript (include quotes in quotation marks "like this"

to

indicate direct quotes from your manuscript), or elaborate on this item by providing additional

information not in the ms, or briefly explain why the item is not applicable/relevant for your study

"Based on intention-to-treat principles, we used mixed effects models accounting for

missing-at-random data and with participant-specific random intercepts to assess the

change in (i) physical activity levels (min/day) and sedentary activity (hr/day) using two time

points (baseline vs. post-intervention), (ii) aerobic fitness levels using three time points

baseline vs. post-intervention vs. follow-up), and (iii) HRQOL scales using two time points

(

(

baseline vs. post-intervention). "

# Does your paper address CONSORT subitem 12b? *

Copy and paste relevant sections from the manuscript (include quotes in quotation marks "like this" to indicate direct quotes from your manuscript), or elaborate on this item by providing additional information not in the ms, or briefly explain why the item is not applicable/relevant for your study

"In addition to quantifying daily time spent in various physical activity intensities at pre- and post-intervention, we used unsupervised data mining methods to further understand physical activity behaviours. We sought to assess behaviours defined by how survivors’ sedentary and MVPA levels were distributed throughout the day, and how these levels evolved with regards to intensity, duration, and frequency of physical activity bouts. Exploring various physical activity bouts plays an important role in understanding how survivors accumulate their physical activity, for example: whether survivors engage in frequent and short bouts of MVPA or less frequent and long bouts of MVPA. To capture these various physical activity intensities, durations, frequencies and bouts, we used the cluster analysis method proposed in earlier work21 to analyse the impact of iBounce on survivors’ physical activity behaviours.

We performed our cluster analysis using R (R Studio), published in Open Science Framework (https://osf.io/ng3tk/). We processed the raw data into one second epoch signal vector magnitude datapoints of activity between 07:00 to 22:00 and then classified each second into a physical activity intensity level (sedentary, light, and MVPA) using activity cut points validated for children aged 8-14 years27. Our cluster analysis grouped participants according to the similarity of their mean daily physical activity and sedentary behaviours to characterise each cluster at pre- and post-intervention. We used principal components analysis to maximise the variance of our accelerometer data, and the elbow method for the total within-cluster sum of square method to confirm the number of acceptable clusters. All participants’ daily physical activity was clustered by eight factors: total time spent in MVPA of ≥3s bouts and ≥30s bouts, total time spent in sedentary activity of ≥60s bouts and ≥300s bouts, frequency of ≥3s and ≥30s MVPA bouts, and frequency of ≥60s and ≥300s sedentary bouts. Informed by Diaz et al.,21 we used at least 3 second bout lengths to represent short MVPA bouts as activity recorded in less than 3 seconds typically represents agitation of the activity tracker, rather than physical activity. We used at least 30 second bout lengths to represent sustained MVPA. Likewise for sedentary activity, we used at least 60 second bout lengths to represent short duration activity, and at least 300 second bout lengths to represent sustained activity. We then compared survivors’ cluster group at baseline and post-intervention to ascertain whether they moved to a more active cluster post-intervention, suggesting behaviour change. "

# X26) REB/IRB Approval and Ethical Considerations [recommended as subheading under "Methods"] (not a CONSORT item)

subitem not at all important

1

2

3

4

5

essential

subitem not at all important

1

2

3

4

5

essential

X26-i) Comment on ethics committee approval

Clear selection

Does your paper address subitem X26-i?

Copy and paste relevant sections from the manuscript (include quotes in quotation marks "like this"

to

indicate direct quotes from your manuscript), or elaborate on this item by providing additional

information not in the ms, or briefly explain why the item is not applicable/relevant for your study

"The study received ethics approval through the Sydney Children’s Hospital Network Human

Research Ethics Committee (HREC/18/SCHN/471) "

x26-ii) Outline informed consent procedures

Outline informed consent procedures e.g., if consent was obtained offline or online (how? Checkbox,

etc.?), and what information was provided (see 4a-ii). See [6] for some items to be included in informed

consent documents.

Does your paper address subitem X26-ii?

Copy and paste relevant sections from the manuscript (include quotes in quotation marks "like this"

to

indicate direct quotes from your manuscript), or elaborate on this item by providing additional

information not in the ms, or briefly explain why the item is not applicable/relevant for your study

"After written informed consent was provided by parents, survivors were enrolled in the

study and provided with account login details to the program. "

subitem not at all important

1

2

3

4

5

essential

RESULTS

a) For each group, the numbers of participants who were randomly

13

assigned, received intended treatment, and were analysed for the primary

outcome

NPT: The number of care providers or centers performing the intervention in each group and the

number of patients treated by each care provider in each center

X26-iii) Safety and security procedures

Safety and security procedures, incl. privacy considerations, and any steps taken to reduce the likelihood

or detection of harm (e.g., education and training, availability of a hotline)

Does your paper address subitem X26-iii?

Copy and paste relevant sections from the manuscript (include quotes in quotation marks "like this"

to

indicate direct quotes from your manuscript), or elaborate on this item by providing additional

information not in the ms, or briefly explain why the item is not applicable/relevant for your study

Your answer

Does your paper address CONSORT subitem 13a?

*

Copy and paste relevant sections from the manuscript (include quotes in quotation marks "like this"

to

indicate direct quotes from your manuscript), or elaborate on this item by providing additional

information not in the ms, or briefly explain why the item is not applicable/relevant for your study

"Of 165 childhood cancer survivors screened for eligibility, 93 did not meet the inclusion

criteria (see Figure 1 for details). We invited the remaining 72 childhood cancer survivors to

our study. Of these 72, 21 actively refused (n=14 were not interested, n=5 were too busy, and

n=2 were unable to travel for assessments) and 21 could not be contacted (i.e., no response

following two phone calls and a voicemail). A total of 30 participants opted into the study.

Participants were a mean age of 10.2 years (SD 1.5), 44% female and on average 5.0 years

(

SD 3.1) from cancer treatment completion. "

13

b) For each group, losses and exclusions after randomisation, together with

reasons

subitem not at all important

1

2

3

4

5

essential

14

a) Dates defining the periods of recruitment and follow-up

Does your paper address CONSORT subitem 13b? (NOTE: Preferably, this is

shown in a CONSORT flow diagram)

*

Copy and paste relevant sections from the manuscript (include quotes in quotation marks "like this"

to

indicate direct quotes from your manuscript), or elaborate on this item by providing additional

information not in the ms, or briefly explain why the item is not applicable/relevant for your study

"Of 165 childhood cancer survivors screened for eligibility, 93 did not meet the inclusion

criteria (see Figure 1 for details). We invited the remaining 72 childhood cancer survivors to

our study. Of these 72, 21 actively refused (n=14 were not interested, n=5 were too busy, and

n=2 were unable to travel for assessments) and 21 could not be contacted (i.e., no response

following two phone calls and a voicemail). A total of 30 participants opted into the study.

Participants were a mean age of 10.2 years (SD 1.5), 44% female and on average 5.0 years

(

SD 3.1) from cancer treatment completion. "

b-i) Attrition diagram

13

Strongly recommended: An attrition diagram (e.g., proportion of participants still logging in or using the

intervention/comparator in each group plotted over time, similar to a survival curve) or other figures or

tables demonstrating usage/dose/engagement.

Does your paper address subitem 13b-i?

Copy and paste relevant sections from the manuscript or cite the figure number if applicable (include

quotes in quotation marks "like this"

to indicate direct quotes from your manuscript), or elaborate on this

item by providing additional information not in the ms, or briefly explain why the item is not

applicable/relevant for your study

Your answer

subitem not at all important

1

2

3

4

5

essential

b) Why the trial ended or was stopped (early

)

14

Does your paper address CONSORT subitem 14a?

*

Copy and paste relevant sections from the manuscript (include quotes in quotation marks "like this"

to

indicate direct quotes from your manuscript), or elaborate on this item by providing additional

information not in the ms, or briefly explain why the item is not applicable/relevant for your study

Mentioned in the methods section - "We recruited participants from Sydney Children’s

Hospital, Australia, between May 2019 and May 2021. Study recruitment was impacted from

March 2020 to May 2021 due to the Covid-19 pandemic, limiting face-to-face consultations

and reducing opportunities for face-to-face communication with families. "

14

a-i) Indicate if critical “secular events” fell into the study period

Indicate if critical “secular events” fell into the study period, e.g., significant changes in Internet

resources available or “changes in computer hardware or Internet delivery resources”

Does your paper address subitem 14a-i?

Copy and paste relevant sections from the manuscript (include quotes in quotation marks "like this"

to

indicate direct quotes from your manuscript), or elaborate on this item by providing additional

information not in the ms, or briefly explain why the item is not applicable/relevant for your study

Your answer

Does your paper address CONSORT subitem 14b?

*

Copy and paste relevant sections from the manuscript (include quotes in quotation marks "like this"

to

indicate direct quotes from your manuscript), or elaborate on this item by providing additional

information not in the ms, or briefly explain why the item is not applicable/relevant for your study

Not applicable to this study.

15)

A table showing baseline demographic and clinical characteristics for each

group

NPT: When applicable, a description of care providers (case volume, qualification, expertise, etc.) and

centers (volume) in each group

subitem not at all important

1

2

3

4

5

essential

For each group, number of participants (denominator) included in each

16)

analysis and whether the analysis was by original assigned groups

Does your paper address CONSORT subitem 15?

*

Copy and paste relevant sections from the manuscript (include quotes in quotation marks "like this"

to

indicate direct quotes from your manuscript), or elaborate on this item by providing additional

information not in the ms, or briefly explain why the item is not applicable/relevant for your study

Demographic and clinical characteristics shown in Table 1.

15-i)

Report demographics associated with digital divide issues

In ehealth trials it is particularly important to report demographics associated with digital divide issues,

such as age, education, gender, social-economic status, computer/Internet/ehealth literacy of the

participants, if known.

Clear selection

Does your paper address subitem 15-i?

*

Copy and paste relevant sections from the manuscript (include quotes in quotation marks "like this"

to

indicate direct quotes from your manuscript), or elaborate on this item by providing additional

information not in the ms, or briefly explain why the item is not applicable/relevant for your study

Demographic and clinical characteristics shown in Table 1.

subitem not at all important

1

2

3

4

5

essential

subitem not at all important

1

2

3

4

5

essential

16-i)

Report multiple “denominators” and provide definitions

Report multiple “denominators” and provide definitions: Report N’s (and effect sizes) “across a range of

study participation [and use] thresholds” [1], e.g., N exposed, N consented, N used more than x times, N

used more than y weeks, N participants “used” the intervention/comparator at specific pre-defined time

points of interest (in absolute and relative numbers per group). Always clearly define “use” of the

intervention.

Clear selection

Does your paper address subitem 16-i?

*

Copy and paste relevant sections from the manuscript (include quotes in quotation marks "like this"

to

indicate direct quotes from your manuscript), or elaborate on this item by providing additional

information not in the ms, or briefly explain why the item is not applicable/relevant for your study

"Of the 23 participants who commenced iBounce, 83% (n=19/23) completed the

intervention.", "Sixteen survivors (n=16/23, 70%) completed the post-intervention fitness test

and returned accelerometers, and 65% (15/23) completed the week 24 follow-up

assessment. "

16-

ii) Primary analysis should be intent-to-treat

Primary analysis should be intent-to-treat, secondary analyses could include comparing only “users”, with

the appropriate caveats that this is no longer a randomized sample (see 18-i).

Does your paper address subitem 16-ii?

Copy and paste relevant sections from the manuscript (include quotes in quotation marks "like this"

to

indicate direct quotes from your manuscript), or elaborate on this item by providing additional

information not in the ms, or briefly explain why the item is not applicable/relevant for your study

Your answer

17

a) For each primary and secondary outcome, results for each group, and the

estimated effect size and its precision (such as 95

%

confidence interval)

subitem not at all important

1

2

3

4

5

essential

b) For binary outcomes, presentation of both absolute and relative effect

17

sizes is recommended

Does your paper address CONSORT subitem 17a?

*

Copy and paste relevant sections from the manuscript (include quotes in quotation marks "like this"

to

indicate direct quotes from your manuscript), or elaborate on this item by providing additional

information not in the ms, or briefly explain why the item is not applicable/relevant for your study

"Survivors did not increase their mean daily MVPA from pre- to post-intervention (mean 39.2

min/day, change -2.5, SE 7.4, 95% CI [-17.6, +12.6], P = .74)."

a-i) Presentation of process outcomes such as metrics of use and intensity of

17

use

In addition to primary/secondary (clinical) outcomes, the presentation of process outcomes such as

metrics of use and intensity of use (dose, exposure) and their operational definitions is critical. This does

not only refer to metrics of attrition (13-b) (often a binary variable), but also to more continuous exposure

metrics such as “average session length”. These must be accompanied by a technical description how a

metric like a “session” is defined (e.g., timeout after idle time) [1] (report under item 6a).

Does your paper address subitem 17a-i?

Copy and paste relevant sections from the manuscript (include quotes in quotation marks "like this"

to

indicate direct quotes from your manuscript), or elaborate on this item by providing additional

information not in the ms, or briefly explain why the item is not applicable/relevant for your study

"Over two-thirds of participants who finished the program (n=13/19, 68%), completed all 10

modules (median=10/10, interquartile range [IQR]=7-10; Figure 2). Activity tracker

engagement was high, with 79% of participants (n=15/19) synchronising their activity

tracker to the program for seven or more iBounce modules. The median number of modules

that activity trackers were worn for, and synchronised to, was 9 modules (IQR=2-10). "

18)

Results of any other analyses performed, including subgroup analyses and

adjusted analyses, distinguishing pre-specified from exploratory

subitem not at all important

1

2

3

4

5

essential

Does your paper address CONSORT subitem 17b?

*

Copy and paste relevant sections from the manuscript (include quotes in quotation marks "like this"

to

indicate direct quotes from your manuscript), or elaborate on this item by providing additional

information not in the ms, or briefly explain why the item is not applicable/relevant for your study

Not applicable to this study.

Does your paper address CONSORT subitem 18?

*

Copy and paste relevant sections from the manuscript (include quotes in quotation marks "like this"

to

indicate direct quotes from your manuscript), or elaborate on this item by providing additional

information not in the ms, or briefly explain why the item is not applicable/relevant for your study

"We assessed changes in physical activity behaviours among the 11 survivors who had

complete accelerometer data at pre- and post-intervention. We retained four principal

components and our cluster analysis resulted in five acceptable cluster groups. Cluster 1

represents the most active group that meets the physical activity guidelines and engages in

the most frequent bouts of MVPA, through to cluster 5 as the least active group with

infrequent and sustained bouts of sedentary behaviours (Table 3). "

18-i)

Subgroup analysis of comparing only users

A subgroup analysis of comparing only users is not uncommon in ehealth trials, but if done, it must be

stressed that this is a self-selected sample and no longer an unbiased sample from a randomized trial

(

).

see 16-iii

Does your paper address subitem 18-i?

Copy and paste relevant sections from the manuscript (include quotes in quotation marks "like this"

to

indicate direct quotes from your manuscript), or elaborate on this item by providing additional

information not in the ms, or briefly explain why the item is not applicable/relevant for your study

Your answer

19)

All important harms or unintended effects in each group

(

for specific guidance see CONSORT for harms

)

subitem not at all important

1

2

3

4

5

essential

Does your paper address CONSORT subitem 19?

*

Copy and paste relevant sections from the manuscript (include quotes in quotation marks "like this"

to

indicate direct quotes from your manuscript), or elaborate on this item by providing additional

information not in the ms, or briefly explain why the item is not applicable/relevant for your study

Not applicable to this study.

Include privacy breaches, technical problems

19-i)

Include privacy breaches, technical problems. This does not only include physical “harm” to participants,

but also incidents such as perceived or real privacy breaches [1], technical problems, and other

unexpected/unintended incidents. “Unintended effects” also includes unintended positive effects [2].

Clear selection

Does your paper address subitem 19-i?

Copy and paste relevant sections from the manuscript (include quotes in quotation marks "like this"

to

indicate direct quotes from your manuscript), or elaborate on this item by providing additional

information not in the ms, or briefly explain why the item is not applicable/relevant for your study

"Over half of participants reported at least one technical difficulty (n=13/23, 57%). The most

commonly reported technical difficulty was synchronisation issues between the Misfit Ray©

activity tracker and the app (n=13/20, 65%). Two of the four participants who dropped out

after commencing the intervention discontinued the study due to these technical difficulties.

All remaining technical issues were resolved through consultations (phone call, SMS or

email) with the study coordinator. Solutions to technical difficulties involved replacing the

activity trackers, sending new batteries, and resetting the modules. "

subitem not at all important

1

2

3

4

5

essential

DISCUSSION

Interpretation consistent with results, balancing benefits and harms, and

22)

considering other relevant evidence

NPT: In addition, take into account the choice of the comparator, lack of or partial blinding, and unequal

expertise of care providers or centers in each group

19-

ii) Include qualitative feedback from participants or observations from

staff/researchers

Include qualitative feedback from participants or observations from staff/researchers, if available, on

strengths and shortcomings of the application, especially if they point to unintended/unexpected effects

or uses. This includes (if available) reasons for why people did or did not use the application as intended

by the developers.

Clear selection

Does your paper address subitem 19-ii?

Copy and paste relevant sections from the manuscript (include quotes in quotation marks "like this"

to

indicate direct quotes from your manuscript), or elaborate on this item by providing additional

information not in the ms, or briefly explain why the item is not applicable/relevant for your study

"Reasons for recommendations included benefits of iBounce as an engaging and

educational program for children, with one survivor highlighting that iBounce may be helpful

for their friends for example, “Because some of my friends aren't that healthy and this would

direct them into the right track” (13-year-old male survivor). Multimedia Appendix S2

summarises survivors’ and parents’ responses to open-ended questions."

# 22-i) Restate study questions and summarize the answers suggested by the data, starting with primary outcomes and process outcomes (use)

Restate study questions and summarize the answers suggested by the data, starting with primary outcomes and process outcomes (use).

1 2 3 4 5

subitem not at all important essential

Clear selection

# Does your paper address subitem 22-i? *

Copy and paste relevant sections from the manuscript (include quotes in quotation marks "like this" to indicate direct quotes from your manuscript), or elaborate on this item by providing additional information not in the ms, or briefly explain why the item is not applicable/relevant for your study

"Distance-delivered physical activity interventions are promising and feasible programs that can offer support and engagement among survivors and families in health behaviours16. The iBounce program appears to be safe, feasible and acceptable, demonstrating moderate opt-in and high retention rates, reflecting that families were interested in physical activity support in survivorship. Our opt-in rate was lower than our feasibility target due to the challenge of study recruitment and potentially the negative impacts of the COVID-19 pandemic. Over 56% of potential participants who were screened did not meet the eligibility criteria, and 58% of eligible participants either declined participation or were unreachable. The challenge of participant recruitment for health behaviour interventions among paediatric oncology is common8,40. However, our challenges may have been exacerbated due to recruitment from a single site and parents not wanting to commit to research interventions due to the unknown risk of COVID-19 for unvaccinated children diagnosed with cancer and their immunocompromised status41. Despite the lower than anticipated opt-in rate, the retention rate and program engagement were high, demonstrating that once survivors expressed interest in iBounce, they were highly receptive to the program. Activity tracker compliance was also lower than the feasibility targets that we set, potentially due to survivors’ preference for a monitor that showed their activity42, and the technical difficulties participants experienced which may have affected adherence. Parent-reported acceptability about the program was also lower than anticipated, potentially due to the technological difficulties which disjointed the program. Some parents reported iBounce to be somewhat burdensome and their qualitative comments were valuable, highlighting the technical difficulties, their dissatisfaction with the Misfit activity tracker and suggestion that iBounce may be better suited to younger children. Despite the technical difficulties experienced by participants, it is encouraging that these issues did not appear to adversely affect participant engagement or the delivery of the program content."

subitem not at all important

1

2

3

4

5

essential

Trial limitations, addressing sources of potential bias, imprecision, and, if

20)

relevant, multiplicity of analyses

subitem not at all important

1

2

3

4

5

essential

22-

ii) Highlight unanswered new questions, suggest future research

Highlight unanswered new questions, suggest future research.

Clear selection

Does your paper address subitem 22-ii?

Copy and paste relevant sections from the manuscript (include quotes in quotation marks "like this"

to

indicate direct quotes from your manuscript), or elaborate on this item by providing additional

information not in the ms, or briefly explain why the item is not applicable/relevant for your study

"Future trials of iBounce should include a “troubleshooting” pamphlet of common technical

issues and solutions to improve participant retention and reduce burden for the study

coordinator." "Future studies should validate or develop simple, home-based functional

assessments for researchers or clinicians to facilitate distance-based exercise testing for

childhood cancer survivors. The development of accurate and reliable home-based

functional assessments has the potential to enable support and reduce the burden for

survivors and the healthcare system."

20-i)

Typical limitations in ehealth trials

Typical limitations in ehealth trials: Participants in ehealth trials are rarely blinded. Ehealth trials often

look at a multiplicity of outcomes, increasing risk for a Type I error. Discuss biases due to non-use of the

intervention/usability issues, biases through informed consent procedures, unexpected events.

Clear selection

# Does your paper address subitem 20-i? *

Copy and paste relevant sections from the manuscript (include quotes in quotation marks "like this" to indicate direct quotes from your manuscript), or elaborate on this item by providing additional information not in the ms, or briefly explain why the item is not applicable/relevant for your study

"Whilst iBounce was available for eligible participants throughout the pandemic, government stay-at-home health orders and social distancing practices limited face-to-face consultations and communication with families, which impacted some pre- and postassessments such as the 6MWT. At the week 24 follow-up, parents assessed their child using the 6MWT, a method that has not yet been validated for parents to administer at home. However, the simplicity, low cost and minimal requirement of equipment for the 6MWT allowed us to educate survivors and families on how to easily assess fitness at home. Another limitation of this study was the ad hoc monitoring of adverse events, which may have led underreporting events, particularly lower grade or non-serious adverse events. Whilst symptoms such as muscle soreness or fatigue reflect common responses to exercise, these low grade adverse events are important to report to provide confidence that unsupervised or home-based exercise is safe after cancer treatment58. Therefore, future distance-delivered physical activity interventions should regularly monitor for adverse events using a standardised approach throughout the intervention to improve adverse event reporting and intervention quality.

Our sample was heterogeneous in terms of cancer diagnoses and included participants who had average fitnesss levels at baseline, which may overrepresent survivors who were more active, potentially biasing the results. Further, most participants in our study were living in metropolitan areas and families from regional or rural regions were underrepresented12. Our sample also included highly educated parents and there was no representation from brain cancer survivors. Our intervention focused on English-speaking participants and future trials of iBounce should consider collaborating with non-English-speaking and culturally and linguistically diverse populations due to their increased barriers to accessing care and poorer health outcomes"

# Generalisability (external validity, applicability) of the trial findings

NPT: External validity of the trial findings according to the intervention, comparators, patients, and care providers or centers involved in the trial

# 21-i) Generalizability to other populations

Generalizability to other populations: In particular, discuss generalizability to a general Internet population, outside of a RCT setting, and general patient population, including applicability of the study results for other organizations

1 2 3 4 5

subitem not at all important essential

subitem not at all important

1

2

3

4

5

essential

OTHER INFORMATION

Registration number and name of trial registry

23)

Does your paper address subitem 21-i?

Copy and paste relevant sections from the manuscript (include quotes in quotation marks "like this"

to

indicate direct quotes from your manuscript), or elaborate on this item by providing additional

information not in the ms, or briefly explain why the item is not applicable/relevant for your study

"Improvements in HRQOL after physical activity have previously been reported in childhood

cancer survivors54-56. Physical activity may improve or maintain aspects of HRQOL

including physical and cognitive function, and reduce cancer-related worry57. The HRQOL

measures in our study were not statistically different from baseline to 12-weeks post-

intervention, potentially due to our small sample size. When compared to normative data in

non-cancer populations, iBounce participants also had similar mean scores compared to

their non-cancer peers, demonstrating high quality of life to begin with."

21-

ii) Discuss if there were elements in the RCT that would be different in a

routine application setting

Discuss if there were elements in the RCT that would be different in a routine application setting (e.g.,

prompts/reminders, more human involvement, training sessions or other co-interventions) and what

impact the omission of these elements could have on use, adoption, or outcomes if the intervention is

applied outside of a RCT setting.

Does your paper address subitem 21-ii?

Copy and paste relevant sections from the manuscript (include quotes in quotation marks "like this"

to

indicate direct quotes from your manuscript), or elaborate on this item by providing additional

information not in the ms, or briefly explain why the item is not applicable/relevant for your study

Your answer

24)

Where the full trial protocol can be accessed, if available

25)

Sources of funding and other support (such as supply of drugs), role of

funders

X27) Conflicts of Interest (not a CONSORT item)

Does your paper address CONSORT subitem 23?

*

Copy and paste relevant sections from the manuscript (include quotes in quotation marks "like this"

to

indicate direct quotes from your manuscript), or elaborate on this item by providing additional

information not in the ms, or briefly explain why the item is not applicable/relevant for your study

Australian New Zealand Clinical Trials Registry (ACTRN12621000259842)

Does your paper address CONSORT subitem 24?

*

Cite a Multimedia Appendix, other reference, or copy and paste relevant sections from the manuscript

(

include quotes in quotation marks "like this"

to indicate direct quotes from your manuscript), or

elaborate on this item by providing additional information not in the ms, or briefly explain why the item is

not applicable/relevant for your study

Yes (ACTRN12621000259842)

Does your paper address CONSORT subitem 25?

*

Copy and paste relevant sections from the manuscript (include quotes in quotation marks "like this"

to

indicate direct quotes from your manuscript), or elaborate on this item by providing additional

information not in the ms, or briefly explain why the item is not applicable/relevant for your study

Not applicable to this study.

subitem not at all important

1

2

3

4

5

essential

About the CONSORT EHEALTH checklist

yes, major changes

yes, minor changes

no

X27-i) State the relation of the study team towards the system being evaluated

In addition to the usual declaration of interests (financial or otherwise), also state the relation of the

study team towards the system being evaluated, i.e., state if the authors/evaluators are distinct from or

identical with the developers/sponsors of the intervention.

Does your paper address subitem X27-i?

Copy and paste relevant sections from the manuscript (include quotes in quotation marks "like this"

to

indicate direct quotes from your manuscript), or elaborate on this item by providing additional

information not in the ms, or briefly explain why the item is not applicable/relevant for your study

Your answer

As a result of using this checklist, did you make changes in your manuscript?

*

What were the most important changes you made as a result of using this

checklist?

Your answer

yes

no

Other:

yes

no

Other:

STOP - Save this form as PDF before you click submit

To generate a record that you filled in this form, we recommend to generate a PDF of this page (on a

Mac, simply select "print" and then select "print as PDF") before you submit it.

When you submit your (revised) paper to JMIR, please upload the PDF as supplementary file.

Don't worry if some text in the textboxes is cut off, as we still have the complete information in our

database. Thank you!

How much time did you spend on going through the checklist INCLUDING

making changes in your manuscript

*

time spent going through checklist was 4 hours

As a result of using this checklist, do you think your manuscript has improved?

*

Would you like to become involved in the CONSORT EHEALTH group?

This would involve for example becoming involved in participating in a workshop and writing an

"Explanation and Elaboration" document

Clear selection

Any other comments or questions on CONSORT EHEALTH

Your answer

Final step: Click submit !

Click submit so we have your answers in our database!

Submit

Clear form

Never submit passwords through Google Forms.

This content is neither created nor endorsed by Google. [Report Abuse](https://docs.google.com/forms/u/0/d/e/1FAIpQLSfZBSUp1bwOc_OimqcS64RdfIAFvmrTSkZQL2-3O8O9hrL5Sw/reportabuse?hl=en_US&source=https://docs.google.com/forms/d/e/1FAIpQLSfZBSUp1bwOc_OimqcS64RdfIAFvmrTSkZQL2-3O8O9hrL5Sw/viewform?hl%3Den_US%26hl%3Den_US%26formkey%3DdGlKd2Z2Q1lNSGQ0THl1azM5MS1aWWc6MA%26rm%3Dfull) - [Terms of Service](https://policies.google.com/terms) - [Privacy Policy](https://policies.google.com/privacy)

[Forms](https://www.google.com/forms/about/?utm_source=product&utm_medium=forms_logo&utm_campaign=forms)
